# Stronger policy required to substantially reduce deaths from PM_2.5_ pollution in China

**DOI:** 10.1038/s41467-020-15319-4

**Published:** 2020-03-19

**Authors:** Huanbi Yue, Chunyang He, Qingxu Huang, Dan Yin, Brett A. Bryan

**Affiliations:** 1 Center for Human-Environment System Sustainability (CHESS), State Key Laboratory of Earth Surface Processes and Resource Ecology (ESPRE), Beijing Normal University, 100875 Beijing, China; 2 School of Natural Resources, Faculty of Geographical Science, Beijing Normal University, 100875 Beijing, China; 3 Centre for Integrative Ecology, Deakin University, VIC3125 Melbourne, Australia

**Keywords:** Environmental impact, Risk factors, Interdisciplinary studies

## Abstract

Air pollution kills nearly 1 million people per year in China. In response, the Chinese government implemented the Air Pollution Prevention and Control Action Plan (APPCAP) from 2013 to 2017 which had a significant impact on reducing PM_2.5_ concentration. However, the health benefits of the APPCAP are not well understood. Here we examine the spatiotemporal dynamics of annual deaths attributable to PM_2.5_ pollution (DAPP) in China and the contribution from the APPCAP using decomposition analysis. Despite a 36.1% increase in DAPP from 2000 to 2017, The APPCAP-induced improvement in air quality achieved substantial health benefits, with the DAPP in 2017 reduced by 64 thousand (6.8%) compared to 2013. However, the policy is unlikely to result in further major reductions in DAPP and more ambitious policies are required to reduce the health impacts of air pollution by 2030 and meet the United Nation’s Sustainable Development Goal 3.

## Abstract (web summary)

Chinese government has implemented the air pollution control measure-the Air Pollution Prevention and Control Action Plan in 2013, whose effects have not been fully studied. Here the authors show that from 2013 to 2017, the plan has achieved substantial public health benefits.

## Introduction

PM_2.5_ pollution refers to particulate matter smaller than 2.5 µm in diameter suspended in the air^1^. Responsible for various respiratory and cardiovascular diseases^2–4^, ambient PM_2.5_ pollution is now the greatest environmental risk factor for human health globally^5,6^. Deaths caused by exposure to ambient PM_2.5_ pollution in a given year, termed deaths attributable to PM_2.5_ pollution (DAPP), is directly influenced by PM_2.5_ concentration in addition to demographic factors and the death rate of diseases (age‐specific and disease‐specific)^6–8^. DAPP in 2017 numbered nearly three million worldwide, three times the deaths caused by AIDS in that year^6,9^. In addition, DAPP is a key indicator of the United Nation’s Sustainable Development Goal (SDG) 3 (Ensure healthy lives and promote well‐being for all at all ages) Target 3.9 (By 2030, substantially reduce the number of deaths and illnesses from hazardous chemicals and air, water and soil pollution and contamination)^10^. As the largest developing country, China is heavily affected by PM_2.5_ pollution resulting from processes of industrialization and urbanization^4,7,11^. To reduce PM_2.5_ pollution, the State Council of China launched the Air Pollution Prevention and Control Action Plan (APPCAP) in 2013 which aimed to lower PM_2.5_ concentration in cities by 10–25% by 2017^12^. Costing about $270 billion, covering more than 300 cities all around China, and spanning the energy, industry, transport, legal and regulatory sectors, the APPCAP is by far the largest air pollution control action ever implemented^13–19^. The Ministry of Ecology and Environment of China declared in 2018 that the PM_2.5_ concentration target of the APPCAP had been achieved^20^. However, the health benefits of PM_2.5_ pollution reduction related to the APPCAP are not well understood and quantifying the impact of this policy is of great significance for guiding future environmental policy needs for achieving SDG Target 3.9.

While some studies have analyzed the PM_2.5_‐related health benefits of the APPCAP in China, the results have varied and conclusions have been conflated. First, changes in DAPP are influenced by several factors^6–8^, yet most previous studies did not differentiate their relative contributions to DAPP. For example, some studies used the decline in DAPP to approximate the health benefits brought by the APPCAP directly^15,21^. Other research tried to isolate the effect of changes in PM_2.5_ concentration on DAPP by assuming other factors were fixed^19,22–24^. Second, inconsistent trends in DAPP have been reported by previous studies (Supplementary Fig. 1). For example, some previous studies have found a declining trend in DAPP after 2013^15,21,22^, while the latest Global Burden of Disease (GBD) report (GBD 2017) found an increase^6,9^. These discrepancies are mainly due to differences in input data, choice of diseases, and the exposure‐response functions used to quantify the relationship between PM_2.5_ concentration and the relative risk of diseases^2,8^ (Supplementary Table 1). A reliable and comprehensive understanding of the spatiotemporal dynamics of DAPP and the relative contributions of the driving factors are urgently needed to clarify the PM_2.5_‐related health benefits of the APPCAP and guide China’s future air pollution policy development to ensure the achievement of SDG Target 3.9.

In this study, we analyzed the effects of PM_2.5_ concentration change on DAPP before the APPCAP (2000–2013) and thereafter (2013–2017). We first estimate DAPP in China from 2000 to 2017 by combining an epidemiological model updated in GBD 2017^6^ and long term PM_2.5_ data^25,26^, and analyze the spatiotemporal dynamics. We then evaluate the relative contributions of changes in population, PM_2.5_ concentration, age structure, and death rate of diseases on DAPP using the decomposition method developed by GBD^6,8,27^. The results show that the APPCAP has achieved substantial public health benefits, reducing DAPP by 64 thousand between 2013 and 2017. Finally, we explore future trends in DAPP to 2030 under two different potential PM_2.5_ pollution control policy scenarios—Trend and Ambitious, combined with the projection of demographic factors and health care. The projections indicate that to make substantial further reductions in DAPP required by SDG 3, stronger policy is still required. Our findings offer important insights for national environmental and public health policy in China and worldwide.

## Results

### Trends in underlying driving factors

**Fig. 1:**
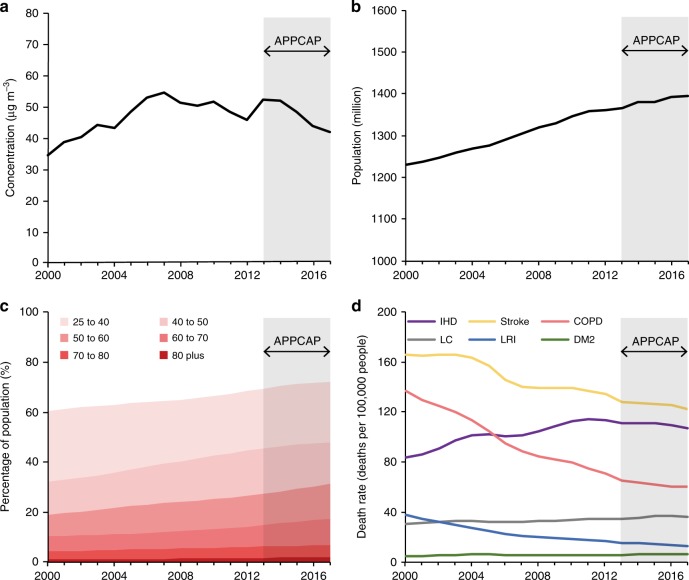
Changes in the driving factors of deaths attributable to PM_2.5_ pollution. **a** population‐weighted PM_2.5_ concentration^59,60^. **b** total population^60,61^. **c** age structure^9^. **d** age‐standardized death rate of diseases^9^. The PM_2.5_ concentration was weighted by population to indicate the overall exposure at the whole of China. IHD, COPD, LC, LRI, and DM2 refer to ischemic heart disease, chronic obstructive pulmonary disease, lung cancer, lower respiratory infection, and diabetes mellitus type 2, respectively. Source data are provided as a Source Data file.

**Fig. 2:**
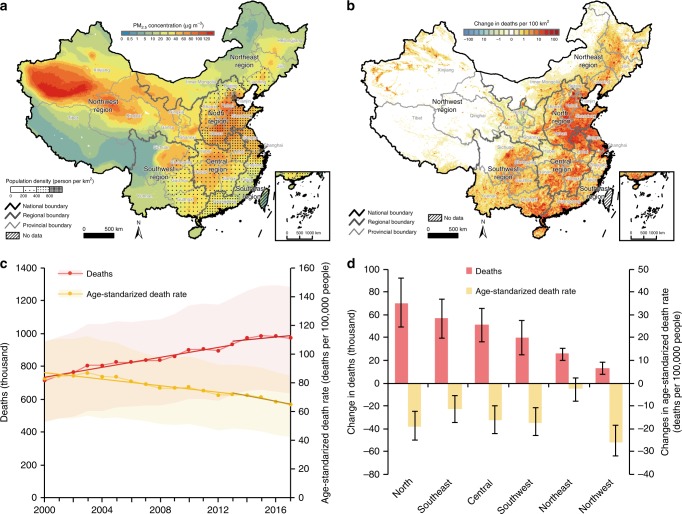
Changes in the deaths attributable to PM_2.5_ pollution (DAPP) and corresponding age‐standardized death rate in China. **a** China’s PM_2.5_ concentration and population density (averages from 2000 to 2017). **b** spatial change in the DAPP between 2000 and 2017. **c** the dynamics of the DAPP and corresponding age‐standardized death rate for China. **d** regional changes in the DAPP and corresponding age‐standardized death rate between 2000 and 2017. Shade and error bars refer to the 90% confidence intervals. Source data are provided as a Source Data file.

Over the last 18 years, the driving factors that influence the DAPP have changed dramatically in China (Figs. 1a, 2a). The population‐weighted PM_2.5_ increased from 2000 to 2007, then showed a fluctuating downward trend. After the establishment of the APPCAP, the population‐weighted PM_2.5_ concentration decreased linearly from 52.5 μg m^−3^ in 2013 to 42.2 μg m^−3^ in 2017 (Fig. 1a). The population maintained a steady trend of growth and aging. The total population and the percentage of old people (60 years or older) increased by 13.2% and 66.0% from 2000 to 2017, respectively (Fig. 1b, c). The changes in age‐standardized death rates varied among diseases, with an overall growth trend in ischemic heart disease, lung cancer, diabetes mellitus type 2, and an overall declining trend in chronic obstructive pulmonary disease, stroke and lower respiratory infection (Fig. 1d).

### Changes in deaths attributable to PM_2.5_ pollution by region

The DAPP in China increased from 714 thousand (458–950) [parentheses contain the 90% confidence interval hereafter] in 2000 to 971 thousand (635–1284) in 2017, increasing 36.1% (35.2–38.6) at an average annual growth rate of 1.8% (1.8–1.9). The annual age‐standardized PM_2.5_ attributable death rate (age‐standardized DAPP per 100,000 people) showed a declining trend, decreasing from 83 (54–109) in 2000 to 65 (42–85) in 2017 (Fig. 2c), this indicator represents the relative health burden without considering the effect of population aging and growth. The increase in DAPP was mainly concentrated in the North region of China, which accounted for about 30% of the total national increase. Increases in DAPP in the other five regions varied from 13 thousand (7–19) in the Northwest region to 57 thousand (39–74) in the Southeast region (Fig. 2d).

Although the DAPP in China continued to increase after the establishment of the APPCAP in 2013, the growth rate declined from 2013 to 2017. The average annual rate of increase in DAPP dropped from 2.1% (1.9–2.4) from 2000 to 2013 to 1.0% (0.3–1.4) from 2013 to 2017. The decreasing trend in PM_2.5_ attributed age‐standardized death rate declined even further, with the average annual rate of change falling from −1.1% (−1.8–−1.3) before APPCAP implementation (2000–2013) to −2.7% (−3.4–−2.2) after (2013–2017). A sequential Mann‐Kendall test also found that the trend in both the variation in DAPP and the corresponding age‐standardized death rate changed significantly in 2013 (Table 1).

**Table 1:** Region‐specific and disease‐specific changes in the deaths and corresponding age‐standardized death rate attributable to PM_2.5_ pollution.

Deaths (thousand)					Age‐standardized death rate (deaths per 100,000 people)			
	Net change between 2000 and 2017	Average annual rate of change (%)			Net change between 2000 and 2017	Average annual rate of change (%)		
		2000–2017	2000–2013	2013–2017		2000–2017	2000–2013	2013–2017
China	258 (177–334)	1.8 (1.8–1.9)	2.1 (1.9–2.4)	1.0^**^ (0.3^**^–1.4^**^)	−19 (−25–−12)	−1.5 (−1.5–−1.4)	−1.1 (−1.3–−0.8)	−2.7^**^ (−3.4^** ^– −2.2^**^)
North	70 (49–90)	1.8 (1.8–1.8)	2.0 (1.9–2.3)	1.0 (0.2–1.5)	−23 (−29–−15)	−1.6 (−1.6–−1.6)	−1.2 (−1.3–−0.9)	−2.9 (−3.7–−2.4)
Southeast	57 (39–74)	2.2 (2.1–2.4)	2.4 (2.1–2.9)	1.6^*^ (1.0^*^–2.0^*^)	−13 (−19–−7)	−1.2 (−1.3–−1.0)	−0.8 (−1.0–−0.3)	−2.5^*^ (−3.2^** ^– −2.1^*^)
Central	52 (36–66)	1.9 (1.9–2)	2.0 (1.9–2.4)	1.5 (1.0–1.8)	−17 (−24–−10)	−1.4 (−1.4–−1.3)	−1.1 (−1.3–−0.8)	−2.1 (−2.6–−1.8)
Southwest	40 (25–55)	1.6 (1.6–1.6)	2.1 (2–2.4)	−0.1^*^ (−1.1^*^–0.6^**^)	−20 (−26–−13)	−1.8 (−1.8–−1.7)	−1.2 (−1.3–−0.9)	−3.6^**^ (−4.7–−2.9^*^)
Northeast	26 (20–30)	2.2 (1.9–2.9)	2.5 (2.1–3.4)	1.3^**^ (1.2^**^–1.4^*^)	−3 (−9 − 2)	−0.3 (−0.6 − 0.3)	0.0 (−0.5–0.8)	−1.2^**^ (−1.3^** ^– −1.1^**^)
Northwest	13 (7–19)	1.1 (1–1.2)	1.4 (1.4–1.5)	0.1 (−0.6–0.6)	−28 (−34–−20)	−2.1 (−2.3–−2.0)	−1.9 (−1.9–−1.8)	−2.9 (−3.7–−2.4)
IHD	145 (101–188)	5.0 (4.9–5.1)	6.1 (5.9–6.4)	1.3^**^ (0.8^**^–1.7^**^)	7 (5 − 8)	2.5 (2.5 − 2.6)	3.9 (3.7 − 4.2)	−1.7 (−2.3–−1.4^**^)
LC	81 (61–100)	5.1 (5–5.2)	6.2 (5.9–6.5)	1.7^**^ (1.1^**^–2.1^**^)	2 (1 − 2)	1.6 (1.6 − 1.8)	2.7 (2.5 − 3.1)	−1.8^**^ (−2.4^** ^– −1.4^**^)
Stroke	61 (32–90)	2.0 (1.9–2.2)	2.2 (2–2.8)	1.1^**^ (0.2^**^–1.6^**^)	−2 (−4–−1)	−0.8 (−0.9–−0.6)	−0.4 (−0.7 − 0.2)	−2.0** (−3.0* – −1.5**)
DM2	18 (14–21)	5.2 (5.2–5.3)	5.3 (5.2–5.4)	4.9 (4.7–5)	0 (0 − 0)	1.5 (1.5 − 1.6)	1.6 (1.6 − 1.8)	1.0 (0.8 − 1.2)
LRI	−22 (−27–−17)	−2.0 (−2.1–−1.9)	−2.2 (−2.4–−1.9)	−1.6 (−2.1–−1.3)	−6 (−7–−5)	−5.4 (−5.5–−5.3)	−5.0 (−5.2–−4.7)	−7.0^**^ (−7.4^** ^– −6.7^**^)
COPD	−26 (−38–−13)	−0.5 (−0.6–−0.4)	−0.8 (−1–−0.4)	0.3 (−0.4–0.8)	−19 (−25–−12)	−4.3 (−4.4–−4.2)	−4.5 (−4.7–−4.1)	−3.9 (−4.5–−3.4)

IHD, COPD, LC, LRI, and DM2 refer to ischemic heart disease, chronic obstructive pulmonary disease, lung cancer, lower respiratory infection, and diabetes mellitus type 2, respectively. Data in the parentheses refer to the 90% confidence intervals.

Asterisks (*) and (**) mean the trend in annual change rate shifted significantly in the year of 2013 at the significance level of 0.1 and 0.05 based on the sequential Mann‐Kendall test.

Following national trends, the increasing trend in DAPP slowed after 2013 at the regional level. The average annual rate of increase in both deaths and age‐standardized death rates during 2013–2017 was lower than that during 2000–2013 for all regions. In addition, a significant break in trend in both deaths and age‐standardized death rates in 2013 was detected by the sequential Mann‐Kendall test in the Southeast, Southwest and Northeast regions (Table 1).

### Changes in deaths attributable to PM_2.5_ pollution by disease

The trend in DAPP from 2000–2017 varied substantially by disease. DAPP from ischemic heart disease and lung cancer increased by 145 thousand (101–188) and 81 thousand (61–100), respectively, accounting for more than 80% of the overall net increase. In addition, DAPP increased in stroke and diabetes mellitus type 2, but decreased in lower respiratory infection and chronic obstructive pulmonary disease (Table 1).

From 2013–2017, the average annual growth rate of deaths and age‐standardized death rates attributable to PM_2.5_ pollution related to ischemic heart disease, lung cancer, stroke and diabetes mellitus type 2 declined relative to that from 2000–2013, in line with the national overall trend. Specifically, the average annual rate of change in DAPP caused by ischemic heart disease declined from 6.1% (5.9–6.4) from 2000 to 2013 to 1.3% (0.8–1.7) from 2013 to 2017. The average annual rate of change in DAPP caused by lung cancer declined from 6.2% (5.9–6.5) from 2000 to 2013 to 1.7% (1.1–2.1) from 2013 to 2017. Furthermore, a significant change in trend occurred in 2013 for both the variation of deaths and age‐standardized death rates caused by ischemic heart disease and lung cancer are detected by the sequential Mann‐Kendall test (Table 1).

### Effects of individual factors

**Fig. 3:**
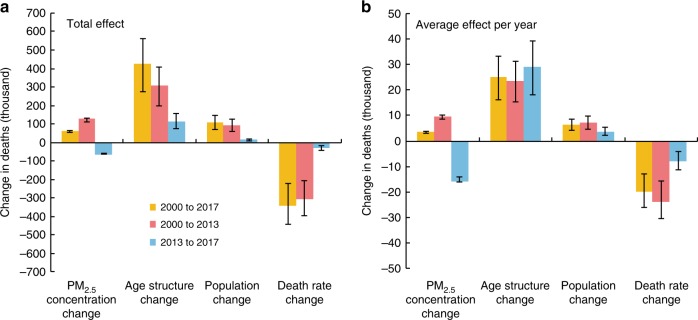
Contributions of different factors to changes in deaths attributable to PM_2.5_ pollution (DAPP) between 2000–2013 and 2013–2017. **a** The total effects in different periods, the sum of the effect caused by different driving factors in each period equals the net change in DAPP. **b** the average effects per year, which equals the total effect divided by the length of each period. Error bars represent 90% confidence intervals. Yellow, red and blue bars represent different periods (2000–2017, 2000–2013, 2013–2017). Source data are provided as a Source Data file.

The net change in DAPP can be decomposed into the effects of four factors, i.e., population, PM_2.5_ concentration, age structure, and death rate of diseases. As a result of changes in PM_2.5_ concentration alone, DAPP in 2017 was 63 thousand (54–67) higher than in 2000. Changes in population and age structure also caused an increase in DAPP of 110 thousand (71–146) and 424 thousand (273–563), respectively in 2017 compared with 2000. Conversely, changes in the death rate of diseases resulted in a decrease of 340 thousand (222–442) in DAPP in 2017 compared with 2000, which partially offset the increases caused by changes in the abovementioned factors (Fig. 3a).

Before and after the establishment of the APPCAP in 2013, the effects of PM_2.5_ concentration change on DAPP showed a contrasting pattern. The DAPP increased by 127 thousand (111–131) between 2000 and 2013 due to changes in PM_2.5_ concentration. By contrast, the DAPP decreased by 64 thousand (57–64) from 2013 to 2017 following the decline in PM_2.5_ concentration after the establishment of the APPCAP (Fig. 3a). The comparison of the average effect per year further revealed the difference in the effects of PM_2.5_ concentration change before and after the APPCAP. Between 2000 and 2013, the DAPP increased by 10 thousand (9–10) per year on average due to changes in PM_2.5_ concentration. While between 2013 and 2017, the decline in PM_2.5_ concentration resulted in an annual average decrease in DAPP of 16 thousand (14–16) per year (Fig. 3b). In terms of the effect of other factors, DAPP increased by 29 thousand (18–39) per year on average between 2013 and 2017 because of changes in age structure. This magnitude is similar to that between 2000 and 2013, due to the stable trend in population aging in the last 18 years. Population growth also resulted in an increase in DAPP, with a growth of 4 thousand (2–5) per year on average between 2013 and 2017, which is lower than that between 2000 and 2013. The effect of changes in death rate declined slightly between 2000–2013 and 2013–2017, resulting in a decrease in DAPP of 24 thousand (16–31) per year on average between 2000 and 2013, and a decrease of 8 thousand (4–11) per year on average between 2013 and 2017. This was mainly due to slowing in the rate of decrease in death rate from the chronic obstructive pulmonary disease from 2013 to 2017^9^. Similar decomposition analysis was conducted for the changes in region‐specific DAPP (Supplementary Fig. 2) and disease‐specific DAPP (Supplementary Fig. 3), and these results were in line with our findings at the national scale.

### Future projection under different control policy scenarios

The DAPP in 2030 under two different air pollution control policy scenarios—Trend and Ambitious was projected (see Methods for details). Both scenarios assume identical future demographic change^28^ and aspirational improvements in health care^29^ to reduce the death rates (Table 2).

**Table 2:** Scenarios for projecting the deaths attributable to PM_2.5_ pollution by 2030.

2030 policy scenario	Description	Population‐weighted PM_2.5_ concentration	Death rate of diseases	Population and age structure
Trend	PM_2.5_ concentration decreases following the current trend of policy.	35 μg m^−3^	30% lower than in 2015, following the Healthy China 2030 Planning Outline^29^.	Estimates from the United Nations under a business as usual trend^28^.
Ambitious	PM_2.5_ concentration decreases substantially by adopting more ambitious air pollution control policy.	10 μg m^−3^		

**Fig. 4:**
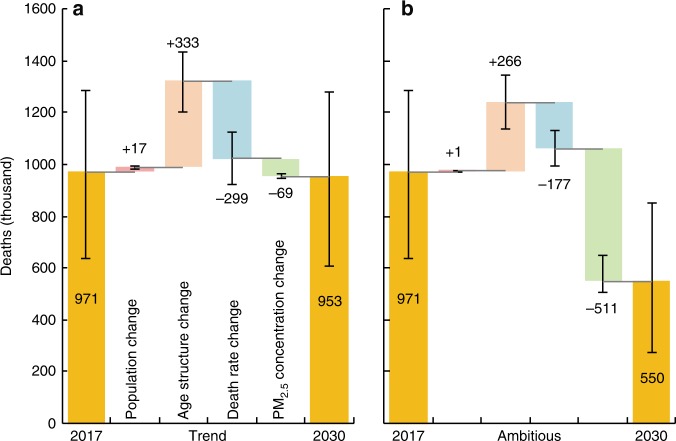
Changes in deaths attributable to PM_2.5_ pollution from 2017 to 2030 under different scenarios. **a** Following the current trend (population‐weighted PM_2.5_ will be 35 μg m^−3^ in 2030). **b** Taking a more ambitious target (population‐weighted PM_2.5_ will be 10 μg m^−3^ in 2030). The effects of changes in population, age structure and death rates are different between the two scenarios because they are jointly affected by the changes in PM_2.5_ concentration (see Methods for details). Error bars represent 90% confidence intervals. Source data are provided as a Source Data file.

Under the Trend scenario, which involves continuing with existing air pollution policy and successfully lowering the population‐weighted PM_2.5_ concentration to 35 μg m^−3^, DAPP was estimated to be 953 thousand (608–1279) in 2030, a net reduction of 18 thousand (5–26) compared to 2017. The changes in PM_2.5_ concentration alone will lead to a reduction in DAPP of 69 thousand (59–74) in 2030 compared to 2017, a reduction of around 7.1% (5.7–9.3) (Fig. 4a). In comparison, under the Ambitious scenario, adopting a stronger air pollution control policy that successfully lowers the population‐weighted PM_2.5_ concentration to 10 μg m^−3^ can substantially reduce DAPP to 550 thousand (275–850) in 2030, a net reduction of 421 thousand (359–433) compared to 2017. Changes in PM_2.5_ concentration alone will lead to a reduction in DAPP of 511 thousand (415–556) in 2030 compared to 2017, a decline of around 52.6% (43.4–65.4) (Fig. 4b). In addition, projected changes in the death rate of diseases will reduce DAPP by 299 thousand (194–397) in 2030 compared to 2017 under the Trend scenario, and 177 thousand (111–243) under the Ambitious scenario. At the same time, population growth will increase the DAPP by 17 thousand (11–22) under the Trend scenario and 1 thousand (−3–4) under the Ambitious scenario, while age structure change will increase the DAPP by 333 thousand (216–443) under the Trend scenario and 266 thousand (163–369) under the Ambitious scenario.

## Discussion

We have quantified the spatiotemporal dynamics in DAPP in China from 2000 to 2017 and further decomposed the relative influence of different factors using the latest data and methods. We found that DAPP had increased from 2000 to 2017, but reductions in PM_2.5_ concentration slowed the growth trend in DAPP after the release of the APPCAP (2013–2017). Future projections under the Trend and Ambitious scenarios indicated that achieving substantial health benefits in 2030 from improvements in air quality requires a more ambitious air pollution control policy.

Compared with previous research, our findings indicated DAPP in China continued the growth trend after the establishment of the APPCAP in 2013. This result is consistent with the latest update of the authoritative GBD 2017^6,9^, but contrasts with some previous studies which indicated that DAPP showed a decreasing trend after 2013^15,21,22^ (Supplementary Fig. 1). The main reason for this discrepancy is that we used the latest input data (e.g., death rate of diseases, age structure) and epidemiological model derived from GBD 2017 (Supplementary Table 1). The health benefits of the APPCAP estimated in our study also differs substantively from existing research^15,21–23^. Although studies agree that APPCAP did avoid substantial DAPP, using the change in overall DAPP to approximate the health benefits of air quality improvement without considering the effects of confounding factors leads to confounded conclusions. For example, this method cannot explain the phenomenon of an increase in DAPP despite air quality improvement. Our decomposition of the effects of PM_2.5_ concentration change relative to the effects of other factors against a counterfactual provides a far more reliable estimate.

The APPCAP reduced deaths from air pollution. DAPP was influenced by population, PM_2.5_ concentration, age structure, and death rate of diseases. From a risk perspective^30^, population reflected the number of people exposed to PM_2.5_ pollution, and PM_2.5_ concentration represented the level of exposure. Exposing more people to a higher level of PM_2.5_ pollution can increase the risk of disease substantially. The age structure and death rates represented the vulnerability of different population groups to air pollution. Compared with demographic factors and death rates, PM_2.5_ concentration is the factor most readily changed via policy to reduce the health risks from air pollution.

The reductions in PM_2.5_ concentrations effected by the APPCAP resulted in 64 thousand (57–64) fewer deaths in DAPP in 2017 compared to 2013. More specifically, the effect of changes in PM_2.5_ concentration on DAPP shifted from positive between 2000 and 2013 when air quality worsened, to negative between 2013 and 2017 when air quality improved. While this is a significant change and ostensibly reflects the positive impact of the APPCAP on health, the reduction in DAPP of 64 thousand (57–64) due to APPCAP‐induced lower PM_2.5_ concentration represented a decline of 6.8% (5.3–9.1) in 2017 relative the 2013 DAPP estimated at 935 thousand (627–1,215). This decline is relatively minor, and the DAPP has continued to increase overall due to the overwhelming influence of other factors. Specifically, the effects of demographic factors (i.e., population and age structure) on DAPP remained positive over time, while the annual negative effect of death rates on DAPP declined from 2013–2017 relative to that from 2000–2013.

Future challenges still remain in the mitigation of DAPP. Although the reduction in PM_2.5_ concentrations caused by the APPCAP had the effect of reducing the DAPP from 2013–2017, the DAPP still increased overall after the APPCAP. Reductions in PM_2.5_ concentrations did not entirely offset the effects of other factors (especially population aging) on DAPP. Material reductions in DAPP, not just the slowing of an increasing trend, is the great challenge that remains to be addressed in China. SDG Target 3.9 of the United Nations call for a substantial reduction in the number of deaths from air pollution^10^. Scenario analysis revealed that DAPP in 2030 will be significantly affected by changes in all four influential factors of population, PM_2.5_ concentration, age structure, and death rate of diseases. Under the Trend scenario, DAPP in 2030 was reduced by 1.9% (0.4–4.1) from 2017 levels under the combined effect of the four driving factors. Lowering the PM_2.5_ concentration to China’s national standard^31^ alone will reduce DAPP by around 7.1% (5.7–9.3), while projected changes in population and age structure will increase DAPP by 1.7% (1.7–1.8) and 34.3% (34.1–34.5), respectively. However, the largest negative effect was due to reductions in the death rates which reduced DAPP by more than 30%. Hence, the impact of future air quality improvements was fairly minor and challenges to achieving a substantial reduction in DAPP by 2030 in alignment with SDG Target 3.9 remain.

By contrast, if China takes a more ambitious air quality target by lowering the PM_2.5_ concentration to the World Health Organization (WHO) standard^32^ of 10 μg m^−3^ by 2030, the DAPP in 2030 will decline by 43.3% (33.8–56.6) compared with that in 2017. While population, aging and death rates are likely to have a similar influence as under the Trend scenario, stronger mitigation of PM_2.5_ emissions under the Ambitious scenario reduced DAPP by 52.6% (43.4–65.4) from 2017 levels. Thus, a more ambitious air pollution policy can help China achieve the intent of SDG target 3.9 of a substantial reduction in DAPP.

China needs to adopt more stringent policies to achieve the WHO standard of 10 μg m^−3^ for PM_2.5_ pollution by 2030 and achieve a substantial reduction in DAPP. In particular, PM_2.5_ emissions from China’s energy system need to be addressed. For example, encouraging clean energy heating and high‐efficiency central heating in the North region can reduce emissions from coal‐burning in rural areas^33–35^. Regulation and management strategies should be enhanced for the main emissions sources. For example, improving the emission‐related laws and regulations for key industries such as steel, iron, and coal‐fired power generation^36,37^. Pollution‐related taxes can be applied to help ensure that emissions will not exceed the standard^38^. Personal behaviors (e.g., inhalation rate and outdoor time) also plays an important role in the exposure of PM_2.5_^22,39^. Strengthening the public awareness of PM_2.5_ pollution and promoting relevant health advisory (e.g., reducing outdoor physical activities during times of heavy pollution) could also be useful for health risk reduction^22,39,40^.

In addition, the joint effects of air pollution abatement and taking climate action towards the achievement of the Paris Agreement and SDG 13^10^ should also be considered in the future. To achieve the target of SDG 13, China plans to lower CO_2_ emissions per unit of GDP by 60–65% and increase the share of non‐fossil fuels in primary energy consumption to around 20% by 2030 from the 2005 level^41^. These efforts are closely related to the air pollution control policies in the energy, transport and industry sectors^42,43^. Hence, stronger air quality control policy is beneficial to achieving both SDG 3 ensuring healthy lives and SDG 13 combating climate change^44^.

A major contribution of this study has been to present a comprehensive and reliable estimate of the health benefit related to the implementation of the APPCAP, which helped explain the discrepancies in previous estimates (Supplementary Fig. 1) and provides new, more accurate results for robust health impact and policy assessment. We used decomposition analysis to quantify the effects of different drivers, to derive more accurate and robust estimates. This allowed for a better understanding of the health benefits of the APPCAP than other comparable research in China^15,21–23^. In addition, our analysis was based on an updated epidemiological model (GBD 2017) which enables a more accurate estimate of DAPP. The latest PM_2.5_ concentration data and population data calibrated by population statistics also improved the accuracy of our estimates. The highly resolved results can provide detailed insights to support decision making and public health management in China. Our findings also have implications for other developing countries that face heavy PM_2.5_ pollution. In the past 25 years, DAPP in developing countries such as India, Pakistan, Bangladesh, and Indonesia have also shown an increasing trend^8,45–48^. Similar efforts should be taken to improve air quality in these countries to reduce DAPP associated with future economic and industrial development.

The results of this study should be considered in light of the following limitations and uncertainties. First, although we presented the 90% confidential intervals of the Integrated Exposure‐Response (IER) function, there are several other uncertainties in our estimation and decomposition of DAPP (e.g., the input data sources, the assessment of exposure, and the use of exposure‐response functions, for details see Supplementary Note 1). Specifically, due to data availability, we used national‐level age structure and death rates^9^ in our estimation. Using provincial‐level death rates (available for three provinces only)^49^ might lead to a different estimate in DAPP. For some regions with a much better healthcare standard (e.g., Beijing), the DAPP was 50% lower compared with DAPP estimated based on national‐level death rates. But for other provinces (e.g., Hebei and Sichuan), the differences were relatively minor (3.2% and 6.1% lower than the main results) (Supplementary Table 2). Quantifying nationwide province‐level death rates will further improve the accuracy of DAPP estimates for China. Regarding the estimates of exposure, we used the annual average PM_2.5_ concentration as a proxy to represent the outdoor exposure, which may overestimate the actual exposure since personal behavior (e.g., inhalation rate and outdoor time) could affect exposure. Considering the impact of personal behavior could be used to refine the assessment of DAPP^39^. Regarding the exposure‐response function, we used the IER from GBD 2017 mainly because it is the most widely used exposure‐response function and the results are comparable across studies. Other exposure‐response functions (e.g., global exposure mortality model, GEMM)^50^ would lead to different estimates of DAPP. But even using the GEMM, the effect of PM_2.5_ concentration change on DAPP also showed a contrasting pattern before and after 2013, which indicated that our key conclusion will still hold (Supplementary Fig. 4).

Second, although changes in PM_2.5_ concentration are predominantly affected by the emission of pollutants^12,15,19^, meteorological conditions such as wind also have some influence^19,51,52^. We did not consider the effects of meteorological factors but directly assumed the air quality improvement from 2013 to 2017 is the result of APPCAP.

Third, our estimation of the health benefit of APPCAP is based on an underlying counterfactual assumption—without the APPCAP, the PM_2.5_ concentration will remain at 2013 levels. The actual situation may be somewhat different, depending on the complex interactions among economic, trade, and weather factors and even individual behavior^33,45,53–55^.

Fourth, the scenarios (i.e., Trend and Ambitious) of future air quality in our study are relatively simple, which cannot reflect the complicated relationships among emissions abatement, climate change and air quality^44,56^. In addition, the decomposition method included only the effects of population, age structure, death rate of disease and PM_2.5_ concentration on DAPP, without considering other underlying socioeconomic and behavioral factors^36,55^. In the future, the estimation of DAPP in China may be improved as new datasets with higher spatial and temporal resolution emerge^57^, and new models capture the effects of other factors, for example by combining new scenario frameworks, emissions inventories and chemical transport models^33,54,58^.

In summary, DAPP in China increased from 2000 to 2017 with more than a quarter of this concentrated in the North region of China. During this 18‐year period, growth in DAPP slowed after the establishment of the APPCAP in 2013. A decline in PM_2.5_ concentration after the release of the APPCAP reduced the DAPP in 2017 by 64 thousand (57–64) or 6.8% (5.3–9.1) compared to that in 2013. However, far greater air pollution abatement efforts are needed in the future. If China takes a more ambitious air quality target and reduces the population‐weighted PM_2.5_ concentration to 10 μg m^−3^ by 2030, the air quality improvement alone will reduce DAPP by 43.3% (33.8–53.6) compared with that in 2017. Thus, China needs to implement a much stronger air pollution mitigation policy to achieve the intent of the United Nation’s SDG Target 3.9 of a substantial reduction in deaths and disease from air pollution.

## Methods

### Gridded PM_2.5_ data

We derived the gridded annual average PM_2.5_ concentration (Estimates of Fine Particulate Matter V4CH02, EFPMV4CH02) from 2000–2016 at a spatial resolution of 0.01 degrees from the Atmospheric Composition Analysis Group^59^. This dataset was generated by combining a chemical transport model, remotely sensed data and monitoring data, which considers both the coverage and accuracy of the estimates for PM_2.5_ concentration (Supplementary Fig. 5)^26^. The detailed descriptions and validation of the above‐mentioned PM_2.5_ data are provided in Supplementary Note 2 and Supplementary Fig. 6. However, gridded data for 2017 were unavailable. Hence, we extrapolated the gridded PM_2.5_ concentration in 2017 based on gridded data in 2016 and the ratio of monitoring data between 2016 and 2017. The monitoring PM_2.5_ concentration was derived from China’s National Urban Air Quality Real Time Publishing Platform^25^. The details of estimating and validating gridded PM_2.5_ concentration for 2017 are provided in Supplementary Note 3 and Supplementary Fig. 7.

### Auxiliary data

Data on the age structure of China’s population from 2000 to 2017, as well as the age‐specific and disease‐specific death rates were obtained from the GBD 2017 dataset^9^. Population distribution data was obtained from the History Database of the Global Environment (HYDE 3.2), which contained the global population from 2000 to 2017 with a spatial resolution of 0.083 degrees^60^. Historical population data covering 284 prefectural‐level cities from 2000 to 2017 (more than 90% of China’s population) were obtained from China City Statistics Yearbooks^61^. Future national‐level population and age structure data under a business as usual scenario was derived from the United Nations^28^. Administrative boundaries were downloaded from the National Geographic Information Public Service Platform of China. The boundaries for the five geographical regions in China were derived from Liu et al. (2005)^62^.

### Estimating deaths attributable to PM_2.5_ pollution

Following the method of the GBD 2017^6^, we estimated DAPP for China from 2000 to 2017 using the comparative risk assessment framework^63^. This framework has been widely used in the health impact assessment conducted by the GBD and WHO at the regional and global scales^6–8^. Its great advantage is that it can estimate deaths caused by multiple risk factors, and the results are comparable across risk factors. In the comparative risk assessment framework, DAPP is determined by four factors: population; age structure; age‐ and disease‐specific death rates; and population attributable fraction (PAF). PAF refers to the proportion of deaths in a population that can be attributed to a certain risk factor, which is determined by the PM_2.5_ concentration and exposure‐response function in this study^8,64^. DAPP in a given year *t* can be calculated as:

1 $${\mathrm{DAPP}}_t\,=\,\mathop {\sum }\limits_{a,{\mathrm{d}}} \left( {{\mathrm{POP}}_t \times {\mathrm{AgeP}}_{a,t} \times {\mathrm{Rate}}_{a,d,t} \times {\mathrm{PAF}}_{a,d,t}} \right)$$

where POP_*t*_ refers to the total population in year *t*; AgeP_*a,t*_ is the proportion of the population with age *a* in year *t*; Rate_*a,d,t*_ is the death rate of disease *d* for people with age *a* in the year *t* and PAF_*a,d,t*_ refers to the proportion of deaths attributed to PM_2.5_ pollution, caused by disease *d*, in a population with age *a* in year *t*. Six kinds of diseases related to PM_2.5_ pollution were considered in this study, including lung cancer, chronic obstructive pulmonary disease, lower respiratory infection, ischemic heart disease, stroke, and diabetes mellitus type 2. Fifteen age groups were included in the equation, i.e., 25–30, 30–35…90–95, and beyond 95 years old. For lower respiratory infection, children less than 5 years old were also considered.

Following the GBD 2017^6^, we calculated the DAPP for China from 2000 to 2017 at 10 km grid cell resolution (Supplementary Fig. 8). The spatial distribution of PAF was calculated based on PM_2.5_ concentration via the IER function updated in GBD 2017^6^. For ischemic heart disease and stroke the IERs were age‐specific, but for other diseases the IERs were uniform across different age groups. Spatial population data was obtained by allocating the prefectural‐level statistical population data^61^ according to the population distribution of the HYDE3.2 dataset. Because only the national death rates and age structure data were published in China, we assumed that the death rates and age structure were homogeneous across the country, following previous studies^65,66^. Detailed formulas and processes are provided in the Supplementary Note 4.

Besides the DAPP which indicates the absolute health effects, we also estimated the age‐standardized death rate related to PM_2.5_ pollution, which refers to the weighted average of the age‐specific death rates (deaths per 100,000 individuals)^67^. The weights were calculated as the percentage of individuals in the corresponding age groups of the standard population set by GBD^68^. Therefore, the age‐standardized death rate attributable to PM_2.5_ can represent the relative health burden by excluding the impacts of demographic changes (i.e., aging and population growth). We then illustrated the changes in total DAPP and PM_2.5_ related age‐standardized death rate for China as a whole, and further analyzed the dynamics of region‐ and disease‐specific DAPP from 2000 to 2017.

### Sequential Mann‐Kendall test

The sequential Mann‐Kendall test^69^ was conducted to detect the abrupt point of change in the trend in DAPP and the corresponding age‐standardized death rate attributable to PM_2.5_ pollution. We used the change rate of annual deaths and age‐standardized death rate in each year as the input to this test. The sequential Mann‐Kendall test calculated two statistics based on a progressive and retrograde series. The point where the progressive and retrograde statistics cross indicates an abrupt change in trend. When either the progressive or retrograde row exceeds specified confidence limits after the crossing point, this abrupt change in trend is considered significant at the corresponding level. Detailed formulas and processes are provided in the Supplementary Note 5.

### Decomposing the effects of individual factors

We dissected the contributions of population, PM_2.5_ concentration, age structure, and death rate of diseases to the change in DAPP using the decomposition method from GBD^5,6,8,27^. It’s worth noting that the PM_2.5_ exposure itself will influence the death rates. In the decomposition, these influences were removed and the effect of death rate change is determined by the standard of health care and other risk factors, independent of the PM_2.5_ exposure. The decomposition method estimates the contribution of factors by sequentially introducing each factor into the DAPP equation. The difference between each consecutive step provides an estimate of the relative contribution of each factor. As the sequence of adding factors also influences the results, we estimated the results under all 24 possible sequences of the four factors. The final estimation of contributions from different factors is the average value of the results for each factor. Detailed equations and processes are shown in Supplementary Fig. 9.

### Projecting future deaths attributable to PM_2.5_ pollution

We projected the DAPP in 2030 by scenario analysis considering China’s commitments to mitigating air pollution and emissions abatement. According to previous reports^13–15,20^, the APPCAP resulted in a substantial reduction in PM_2.5_ concentration. Following the trend after the release of the APPCAP, The State Council of China further proposed the Three‐year action plan aims for cleaner air in 2018, which stipulated that the emissions of sulfur dioxide and nitrogen oxides should decline at least by 15% from 2015 levels by 2020^70^. In addition, China planned to lower its carbon dioxide emissions to combat climate change. In the Nationally Determined Contributions for United Nations Framework Convention on Climate Change released in 2015, China planned to increase the share of non‐fossil fuel in primary energy consumption by around 20% from 2005 level by 2030^41^ (which was 14% in 2017)^71^. Consistent with this policy background, we formulated two scenarios depicting the trend of PM_2.5_ concentration from 2017 to 2030—Trend and Ambitious. In the Trend scenario, the reduction in emissions follows the current trend where the population‐weighted PM_2.5_ concentration is expected to achieve China’s stated air quality standard^31^ of 35 μg m^−3^ by 2030. In the Ambitious scenario, we assumed that China will take a more ambitious policy and the population‐weighted PM_2.5_ concentration will achieve WHO’s air quality guideline^32^ of 10 μg m^−3^ by 2030. In addition, of the four components influencing DAPP, population growth and population aging cannot be effectively altered by policy intervention in the short term. Hence, we assumed that population and age structure will follow the business as usual estimates of the United Nations^28^. The death rate of diseases can feasibly be changed by improving health care. Following the target of lowering the death rate from chronic disease by 30% from 2015 to 2030 set in the Healthy China 2030 Planning Outline in 2016^29^, we assumed that the death rates of all PM_2.5_‐related diseases in 2030 will be 30% lower than in 2015. We then estimated the DAPP in 2030 and then quantified the influence of each factor.

### Reporting summary

Further information on research design is available in the Nature Research Reporting Summary linked to this article.

## Supplementary information

Supplementary Information

Reporting Summary

## Data Availability

All the data created in this study are openly available at Github repositories with the identifier https://github.com/yuehuanbi/air-pollution. The source data underlying Figs. 1–4 are provided as a Source Data file. Other data are available from the corresponding author upon reasonable request.

## Source Data

Source Data

## Code availability

We used the integrated exposure-response function updated in the Global Burden of Disease 2017 to estimate the relative risk caused by PM_2.5_ exposure. The detailed function is accessible to all users at http://ghdx.healthdata.org/record/ihme-data/gbd-2017-burden-risk-1990-2017. Other data processing in this study are conducted at ArcGIS and Microsoft Excel, detailed flowchart was described in the Supplementary Information.

## References

[1] Kaiser J (2005). Mounting evidence indicts fine-particle pollution. Science.

[2] Burnett R (2014). An Integrated Risk Function for Estimating the Global Burden of Disease Attributable to Ambient Fine Particulate Matter Exposure. Environ. Health Perspect..

[3] Bowe B (2018). The 2016 global and national burden of diabetes mellitus attributable to PM2.5 air pollution. Lancet Planet. Health.

[4] West J (2016). “What We Breathe Impacts Our Health: improving Understanding of the Link between Air Pollution and Health”. Environ. Sci. Technol..

[5] GBD 2016 Risk Factors Collaborators. (2017). Global, regional, and national age-sex specific mortality for 264 causes of death, 1990-2016: a systematic analysis for the Global Burden of Disease Study 2016. Lancet.

[6] GBD 2017 Risk Factor Collaborators. (2018). Global, regional, and national comparative risk assessment of 84 behavioural, environmental and occupational, and metabolic risks or clusters of risks for 195 countries and territories, 1990–2017: a systematic analysis for the Global Burden of Disease Study 2017. Lancet.

[7] World Health Organization. *Ambient air pollution: a global assessment of exposure and burden of disease*. http://www.who.int/phe/publications/air-pollution-global-assessment/en/ (2016).

[8] Cohen, A. et al. Estimates and 25-year trends of the global burden of disease attributable to ambient air pollution: an analysis of data from the Global Burden of Diseases Study 2015. *Lancet* **389**, 1907–1918 (2017). 10.1016/S0140-6736(17)30505-6 PMC5439030 28408086

[9] Institute for Health Metrics and Evaluation. *GBD Compare*. https://vizhub.healthdata.org/gbd-compare/ (2018).

[10] United Nations. *Sustainable Development Goals*. https://sustainabledevelopment.un.org/ (2015).

[11] He C, Han L, Zhang R (2016). More than 500 million Chinese urban residents (14% of the global urban population) are imperiled by fine particulate hazard. Environ. Pollut..

[12] The State Council of China. *Air Pollution Prevention and Control Action Plan*. http://www.gov.cn/jrzg/2013-09/12/ (2013).

[13] The State Council of China. *Accessment Method of Air Pollution Prevention and Control Action Plan*. http://www.gov.cn/zhengce/content/2014-05/27/content_8830.htm (2014).

[14] Greenstone, M. & Schwarz, P. *Is China Winning its War on Pollution?* https://aqli.epic.uchicago.edu/wp-content/uploads/2018/08/China-Report.pdf (2018).

[15] Huang J, Pan X, Guo X, Li G (2018). Health impact of China’s Air Pollution Prevention and Control Action Plan: an analysis of national air quality monitoring and mortality data. Lancet Planet. Health.

[16] Editorial. (2019). Cleaner air for China. Nat. Geosci..

[17] Clean Air Alliance of China. *Financing investment and impact of implementing Air Pollution Prevention and Control Action Plan (2013-2017)*. http://www.cleanairchina.org/file/loadFile/110.html (2015).

[18] Ministry of Ecology and Environment of China. *China will invest 1.7 trillion to control air pollution*. http://zfs.mee.gov.cn/hjjj/hjjjzcywxz/201412/t20141201_292210.shtml (2013).

[19] Zhang, Q. et al. Drivers of improved PM2.5 air quality in China from 2013 to 2017. *Proc. Natl Acad. Sci. USA* **116**, 24463–24469 (2019). 10.1073/pnas.1907956116 PMC6900509 31740599

[20] Ministry of Ecology and Environment of China. *Final Assessment of the Implementation of Air Pollution Prevention and Control Action Plan*. http://www.mee.gov.cn/xxgk2018/xxgk/xxgk15/201806/t20180601_630217.html (2017).

[21] Lu, X. et al. Analysis of the adverse health effects of PM2.5 from 2001 to 2017 in China and the role of urbanization in aggravating the health burden. *Sci. Total Environ.* **652**, 683–695 (2019). 10.1016/j.scitotenv.2018.10.140 30380476

[22] Zou B (2019). Air pollution intervention and life-saving effect in China. Environ. Int..

[23] Zheng, Y. et al. Air quality improvements and health benefits from China’s clean air action since 2013. *Environ. Res. Lett.* **12**, 10.1088/1748-9326/aa1088a1032 (2017).

[24] Xue T (2019). Rapid improvement of PM2.5 pollution and associated health benefits in China during 2013–2017. Sci. China Earth Sci..

[25] Ministry of Ecology and Environment of China. *China’s National Urban Air Quality Real Time Publishing Platform*. http://www.mee.gov.cn/hjzl/dqhj/qgkqzlssfb/ (2013).

[26] van Donkelaar A, Martin RV, Li C, Burnett RT (2019). Regional Estimates of Chemical Composition of Fine Particulate Matter Using a Combined Geoscience-Statistical Method with Information from Satellites, Models, and Monitors. Environ. Sci. Technol..

[27] Gupta, P. *Standardization and decomposition of rates: a user’s manual*. https://play.google.com/store/books/details?id=8wjPjfHpttQC&rdid=book-8wjPjfHpttQC&rdot=1 (1993).

[28] United Nations. World Population Prospects 2019. https://population.un.org/wpp/Download/Standard/Population/ (2019).

[29] The State Council of China. *Healthy China 2030 Planning Outline*. http://www.gov.cn/zhengce/2016-10/25/content_5124174.htm (2016).

[30] Intergovernmental Panel on Climate Change. *Managing the risks of extreme events and disasters to advance climate change adaptation*. https://www.ipcc.ch/report/managing-the-risks-of-extreme-events-and-disasters-to-advance-climate-change-adaptation/ (2012). 10.1136/jech-2012-201045 22766781

[31] Ministry of Environment Protection of China. *Ambient Air Quality Standards*. http://kjs.mee.gov.cn/hjbhbz/bzwb/dqhjbh/dqhjzlbz/201203/W020120410330232398521.pdf (2012).

[32] World Health Organization. *WHO Air quality guidelines for particulate matter, ozone, nitrogen dioxide and sulfur dioxide*. http://apps.who.int/iris/bitstream/10665/69477/1/WHO_SDE_PHE_OEH_06.02_eng.pdf (2005). 34662007

[33] Shen, H. et al. Urbanization-induced population migration has reduced ambient PM2.5 concentrations in China. *Sci. Adv.* **3**, 10.1126/sciadv.1700300 (2017). 10.1126/sciadv.1700300 PMC5517109 28776030

[34] Chen Y, Ebenstein A, Greenstone M, Li H (2013). Evidence on the impact of sustained exposure to air pollution on life expectancy from China’s Huai River policy. Proc. Natl Acad. Sci. USA.

[35] Ebenstein A, Fan MY, Greenstone M, He GJ, Zhou MG (2017). New evidence on the impact of sustained exposure to air pollution on life expectancy from China’s Huai River Policy. Proc. Natl Acad. Sci. USA.

[36] Guan, D. et al. The socioeconomic drivers of China’s primary PM 2.5 emissions. *Environ. Res. Lett.* **9**, 10.1088/1748-9326/1089/1082/024010 (2014).

[37] GBD MAPS Working Group. *Burden of Disease Attributable to Coal-Burning and Other Air Pollution Sources in China*. https://www.healtheffects.org/publication/burden-disease-attributable-coal-burning-and-other-air-pollution-sources-china (2016).

[38] Dissou Y, Karnizova L (2016). Emissions cap or emissions tax? A multi-sector business cycle analysis. J. Environ. Econ. Manag..

[39] Zou, B. et al. Efforts in reducing air pollution exposure risk in China: State versus individuals. *Environ. Int.* **137**, 10.1016/j.envint.2020.105504 (2020). 10.1016/j.envint.2020.105504 32032774

[40] National Health Commission of China. *Guideline of protecting the population’s health from air pollution (haze)*. http://www.nhc.gov.cn/jkj/s7934td/201912/63275bbd448543a599e3b1b5a7d2f32e.shtml (2019).

[41] National Development and Reform Commission of China. *China’s First Nationally Determined Contributions*. https://www4.unfccc.int/sites/NDCStaging/Pages/All.aspx (2015).

[42] Guan D (2018). Structural decline in China’s CO2 emissions through transitions in industry and energy systems. Nat. Geosci..

[43] Liu Q, Lei Q, Xu H, Yuan J (2018). China’s energy revolution strategy into 2030. Resour., Conserv. Recycling.

[44] Sandalow, D. *Guide to Chinese Climate Policy 2019*. https://energypolicy.columbia.edu/research/report/guide-chinese-climate-policy (2019).

[45] Fuller GW, Font A (2019). Keeping air pollution policies on track. Science.

[46] India State-Level Disease Burden Initiative Air Pollution Collaborators. (2018). The impact of air pollution on deaths, disease burden, and life expectancy across the states of India: the Global Burden of Disease Study 2017. Lancet Planet. Health.

[47] Apte J, Brauer M, Cohen A, Ezzati M, Pope A (2018). Ambient PM2.5 Reduces Global and Regional Life Expectancy. Environ. Sci. Technol. Lett..

[48] Yue, H., Huang, Q., He, C., Zhang, X. & Fang, Z. Spatiotemporal patterns of global air pollution: a multi-scale landscape analysis based on dust and sea-salt removed PM2.5 data. *J. Clean. Production* **252**, 10.1016/j.jclepro.2019.119887 (2020).

[49] Zhou M (2019). Mortality, morbidity, and risk factors in China and its provinces, 1990–2017: a systematic analysis for the Global Burden of Disease Study 2017. Lancet.

[50] Burnett R (2018). Global estimates of mortality associated with long-term exposure to outdoor fine particulate matter. Proc. Natl Acad. Sci. USA.

[51] Cheng J (2019). Dominant role of emission reduction in PM2.5 air quality improvement in Beijing during 2013–2017: a model-based decomposition analysis. Atmos. Chem. Phys..

[52] Cai W, Li K, Liao H, Wang H, Wu L (2017). Weather conditions conducive to Beijing severe haze more frequent under climate change. Nat. Clim. Change.

[53] Lanzi, E. & Dellink, R. *Economic interactions between climate change and outdoor air pollution*. https://www.oecd-ilibrary.org/content/paper/8e4278a2-en (2019).

[54] Zhang Q (2017). Transboundary health impacts of transported global air pollution and international trade. Nature.

[55] Huang C (2017). Potential Cardiovascular and Total Mortality Benefits of Air Pollution Control in Urban China. Circulation.

[56] West JJ (2013). Co-benefits of mitigating global greenhouse gas emissions for future air quality and human health. Nat. Clim. Change.

[57] Wei, J. et al. Estimating 1-km-resolution PM2.5 concentrations across China using the space-time random forest approach. *Remote Sens. Environ.* **231**, 10.1016/j.rse.2019.111221 (2019).

[58] Rao S (2017). Future air pollution in the Shared Socio-economic Pathways. Glob. Environ. Change.

[59] Atmospheric Composition Analysis Group. *Surface PM2.5*. http://fizz.phys.dal.ca/~atmos/martin/?page_id=140 (2019).

[60] Klein Goldewijk K, Beusen A, Doelman J, Stehfest E (2017). Anthropogenic land use estimates for the Holocene–HYDE 3.2. Earth Syst. Sci. Data.

[61] National Bureau of Statistics of China. *China City Statistics Yearbooks*. http://navi.cnki.net/knavi/YearbookDetail?pcode=CYFD&pykm=YINFN (2018).

[62] Liu J (2005). Spatial and temporal patterns of China’s cropland during 1990–2000: an analysis based on Landsat TM data. Remote Sens. Environ..

[63] Murray, C., Ezzati, M., Lopez, A., Rodgers, A. & Vander Hoorn, S. Comparative quantification of health risks: conceptual framework and methodological issues. *Population Health Metrics* **1**, 10.1186/1478-7954-1181-1181 (2003). 10.1186/1478-7954-1-1 PMC156894 12780936

[64] Rockhill B, Newman B, Weinberg C (1998). Use and misuse of population attributable fractions. Am. J. Public Health.

[65] Liu M (2017). Spatial and temporal trends in the mortality burden of air pollution in China: 2004-2012. Environ. Int..

[66] Silva, R. et al. Global premature mortality due to anthropogenic outdoor air pollution and the contribution of past climate change. *Environ. Res. Lett.* **8**, 10.1088/1748-9326/1088/1083/034005 (2013).

[67] World Health Organization. *Age Standardization of Rates: a new WHO Standard.* https://www.who.int/healthinfo/paper31.pdf (2001).

[68] GBD 2017 Causes of Death Collaborators. (2018). Global, regional, and national age-sex-specific mortality for 282 causes of death in 195 countries and territories, 1980-2017: a systematic analysis for the Global Burden of Disease Study 2017. Lancet.

[69] World Meteorological Organization. *On the statistical analysis of series of observations*. https://library.wmo.int/index.php?lvl=notice_display&id=7427#.XWUz2egzaM8 (1991).

[70] The State Council of China. *Three-year Action Plan Aims for Cleaner Air*. http://english.gov.cn/policies/latest_releases/2018/07/03/content_281476207708632.htm (2018).

[71] Ministry of Ecology and Environment of China. *China’s Policies and Actions for Addressing Climate Change (2017)*. http://english.mee.gov.cn/Resources/Reports/reports/201812/P020181203550137987019.pdf (2018).

