# Supplementary material for: Stronger policy required to substantially reduce deaths from PM2.5 pollution in China
Source: Nat Commun. 2020 Mar 19;11:1462. doi: 10.1038/s41467-020-15319-4 (PMC7081205; doi:10.1038/s41467-020-15319-4)
Supplement: Supplementary file 1 — Supplementary Information [file 41467_2020_15319_MOESM1_ESM.pdf]

Supplementary Information

Stronger policy required to substantially reduce deaths from $PM_{2.5}$ pollution in China

Yue et al.

Contents

Supplementary Figure 1. Comparing deaths attributable to $PM_{2.5}$ pollution between our research and previous studies.

Supplementary Figure 2. Region-specific contributions of different factors to changes in deaths attributable to $PM_{2.5}$ pollution between 2000–2013 and 2013–2017.

Supplementary Figure 3. Disease-specific contributions of different factors to changes in deaths attributable to $PM_{2.5}$ pollution between 2000–2013 and 2013–2017.

Supplementary Figure 4. Results estimated based on the global exposure mortality model.

Supplementary Figure 5. Time-span of $PM_{2.5}$ concentration data used in this study.

Supplementary Figure 6. Comparison between the gridded $PM_{2.5}$ data and air quality monitoring data.

Supplementary Figure 7. Comparison between the gridded and estimated population-weighted $PM_{2.5}$ concentration in 2016.

Supplementary Figure 8. Flow chart for estimating deaths attributable to $PM_{2.5}$ pollution.

Supplementary Figure 9. Framework of the decomposition analysis.

Supplementary Table 1. Comparative summary of study area, exposure-response functions, and input data between previous studies.

Supplementary Table 2. Comparison between deaths attributable to $PM_{2.5}$ pollution (DAPP) estimated based on national death rates and provincial death rates in 2017.

Supplementary Note 1. Detailed uncertainty analysis.

Supplementary Note 2. Detailed description of $PM_{2.5}$ concentration data sources.

Supplementary Note 3. Estimating gridded $PM_{2.5}$ concentration for 2017.

Supplementary Note 4. Calculating deaths attributable to $PM_{2.5}$ pollution at the pixel scale.

Supplementary Note 5. Detailed formula of sequential Mann-Kendall test.

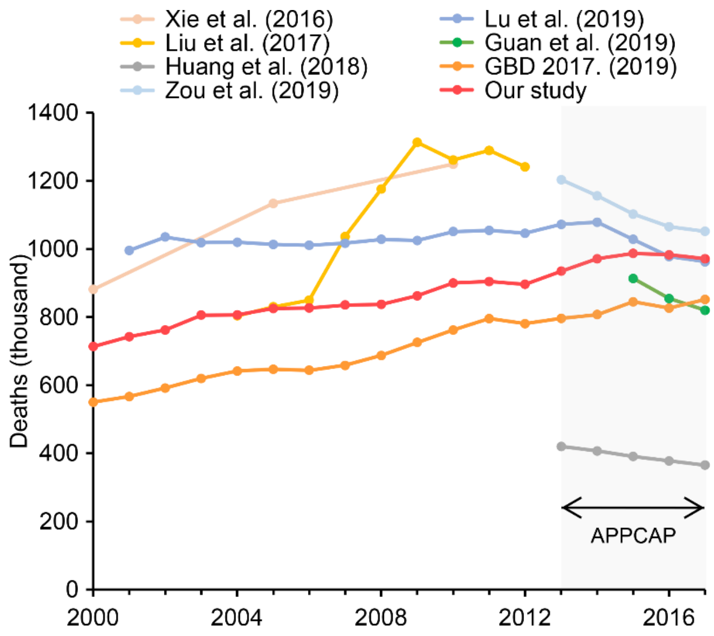

Supplementary Figure 1. Comparing deaths attributable to $PM_{2.5}$ pollution between our research and previous studies. Detailed summary of the diseases considered, exposure-response functions, and input data for these studies can be found in Supplementary Table 1.

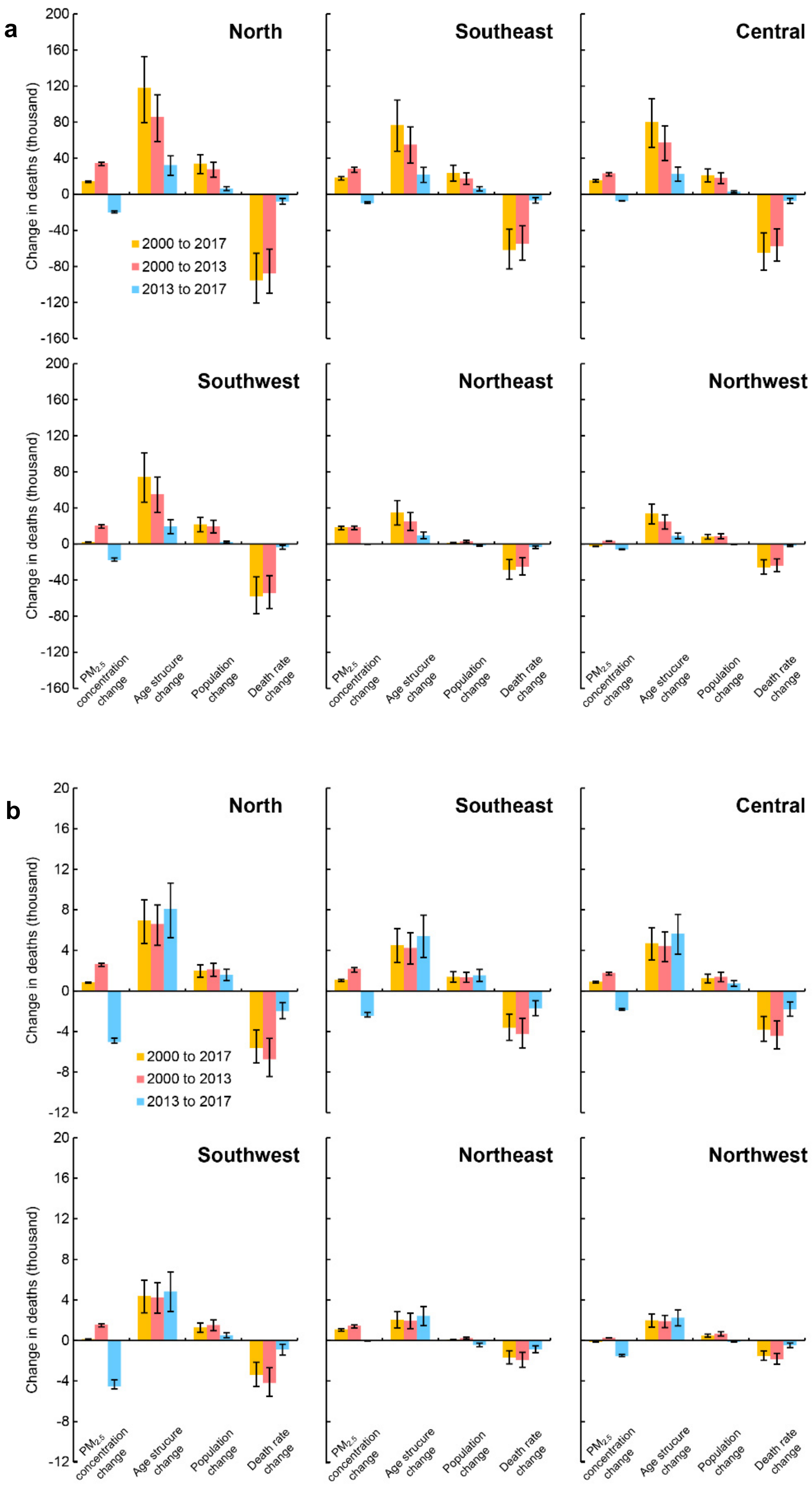

Supplementary Figure 2. Region-specific contributions of different factors to changes in deaths attributable to $PM_{2.5}$ pollution between 2000–2013 and 2013–2017. a, the total effects in different period. **b,** the average effects per year. Error bars refer to the 90% confidence intervals.

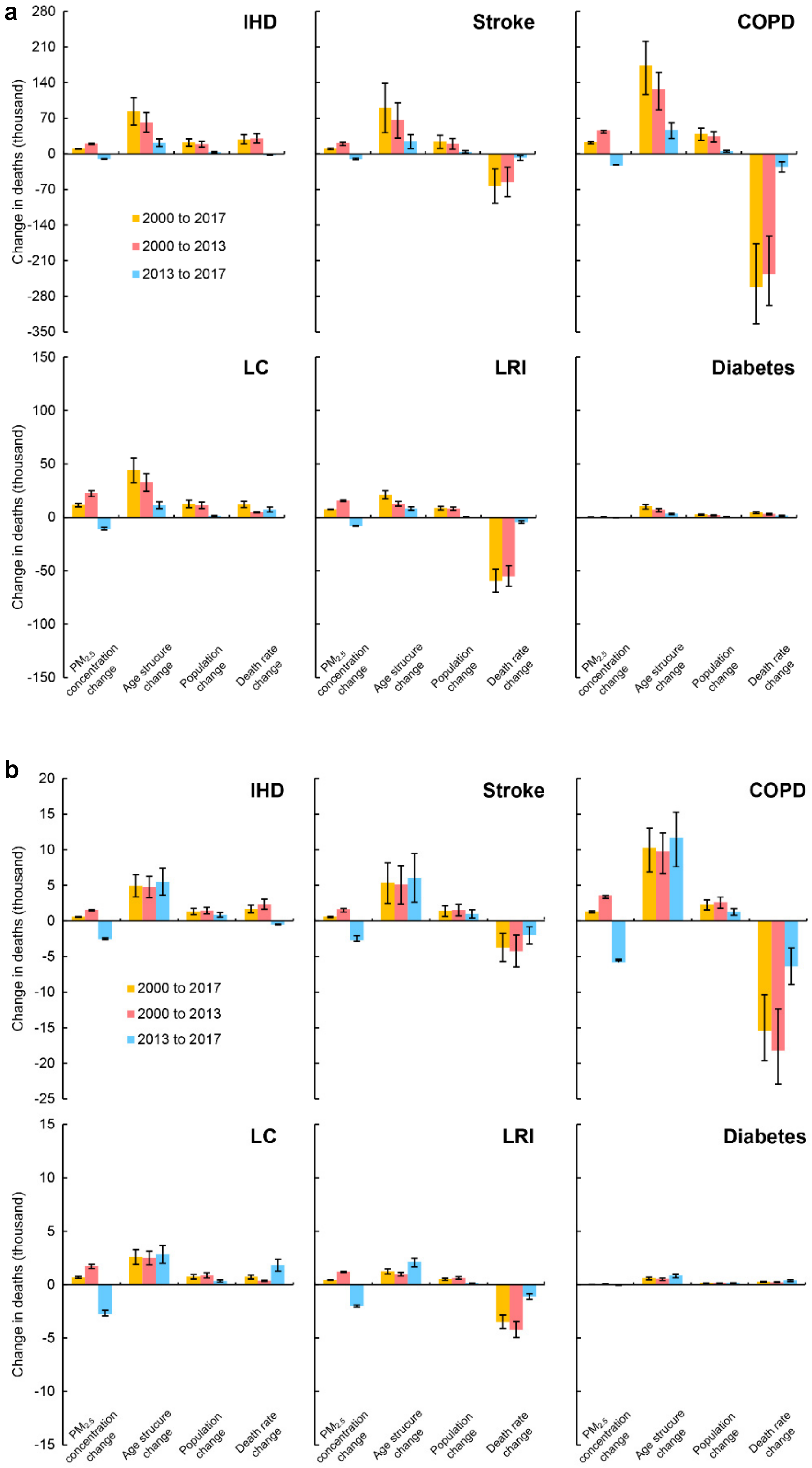

Supplementary Figure 3. Disease-specific contributions of different factors to changes in deaths attributable to $PM_{2.5}$ pollution between 2000–2013 and 2013–2017. a, the total effects in different period. **b,** the average effects per year. Error bars refer to the 90% confidence intervals. IHD, COPD, LC, LRI, and DM2 refer to the ischemic heart disease, chronic obstructive pulmonary disease, lung cancer, lower respiratory infection, and diabetes mellitus type 2, respectively.

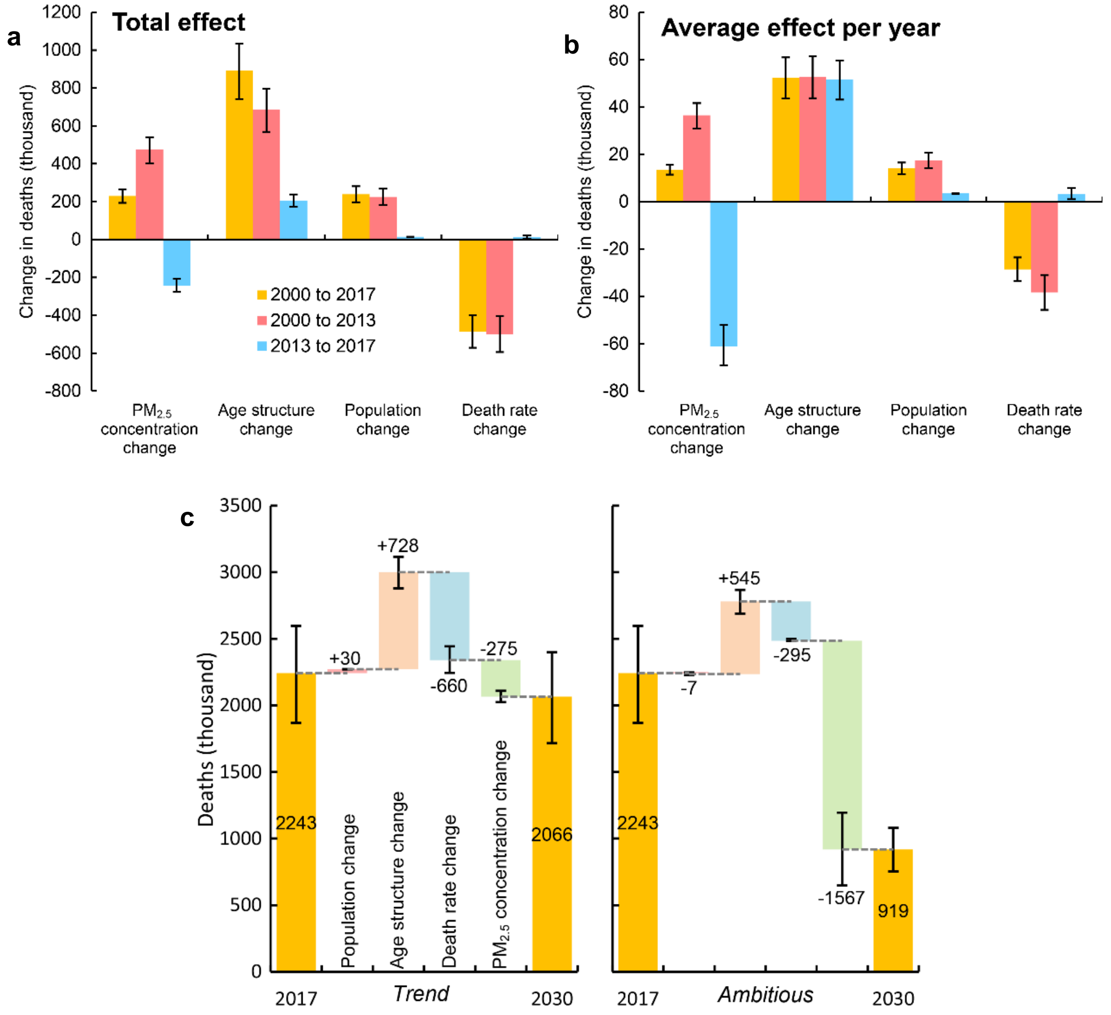

Supplementary Figure 4. Results estimated based on the global exposure mortality model. a, the total effects of decomposition from 2000–2017. **b,** the average effects per year of decomposition from 2000–2017. **c,** Changes in deaths attributable to $PM_{2.5}$ pollution from 2016 to 2030 under different scenarios. Error bars refer to the 95% confidence intervals.

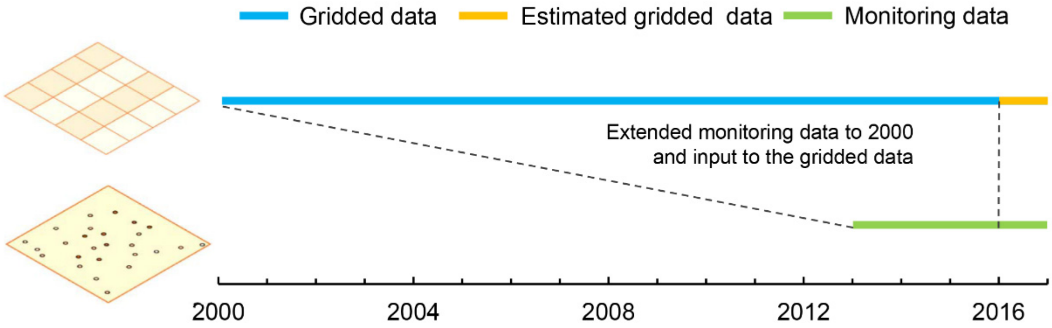

Supplementary Figure 5. Time-span of $PM_{2.5}$ concentration data used in this study. The gridded $PM_{2.5}$ data was calibrated by monitoring data in China. Because the monitoring network of $PM_{2.5}$ concentration in China was established in 2013, the data provider extends these values back to 2000 based on remotely sensed $PM_{2.5}$ data. The estimated gridded data was extrapolated by monitoring data, because gridded $PM_{2.5}$ concentration data was unavailable for the year 2017.

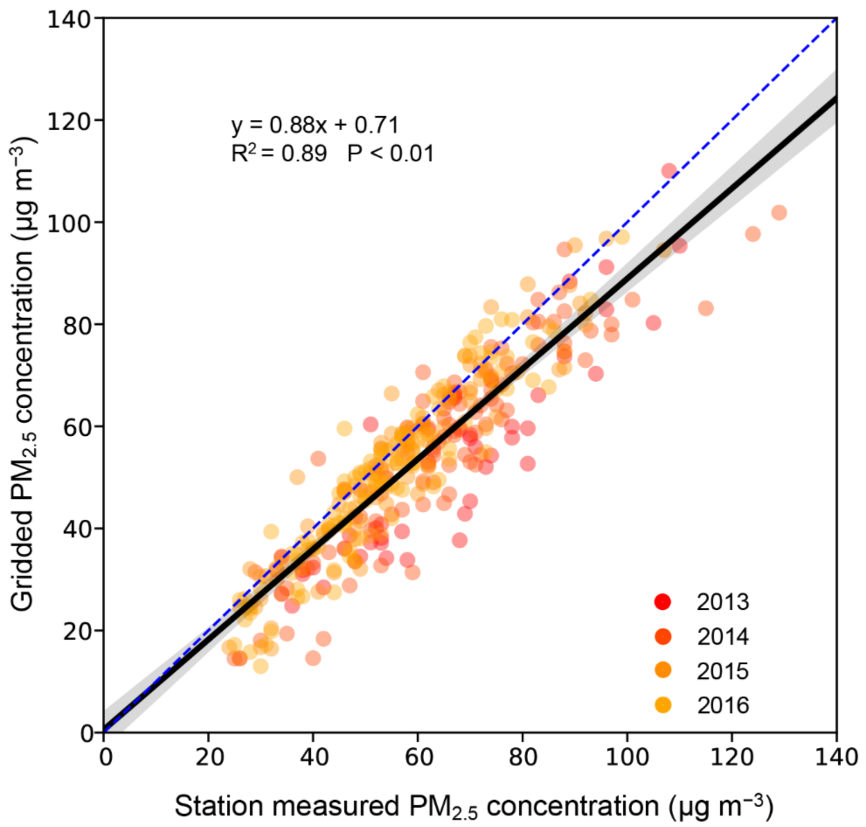

Supplementary Figure 6. Comparison between the gridded $PM_{2.5}$ data and air quality monitoring data.

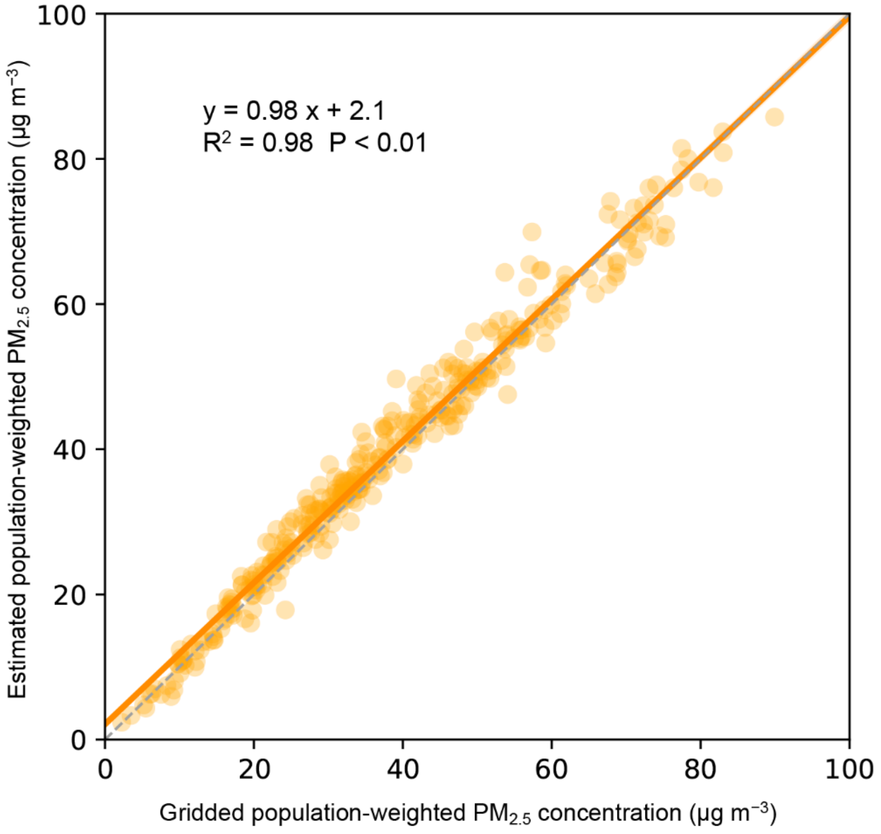

Supplementary Figure 7. Comparison between the gridded and estimated population-weighted $PM_{2.5}$ concentration in 2016.

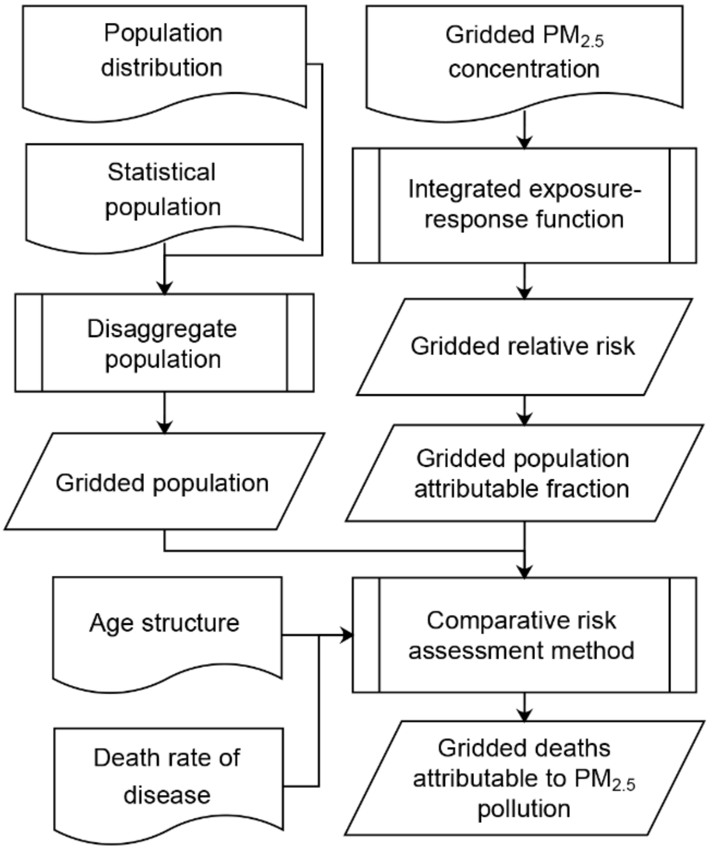

Supplementary Figure 8. Flow chart for estimating deaths attributable to $PM_{2.5}$ pollution.

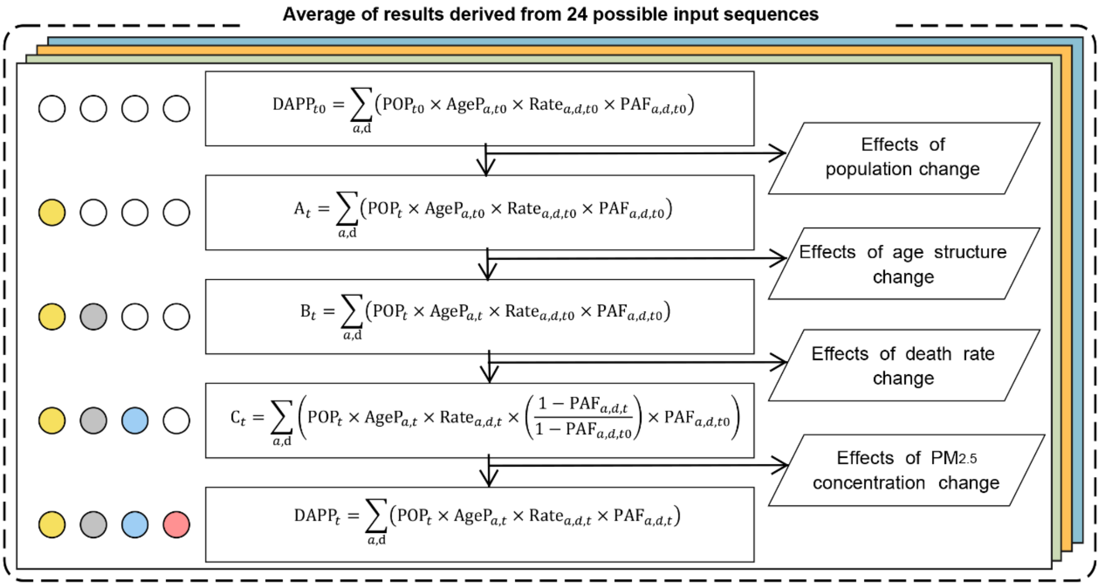

Supplementary Figure 9. Framework of the decomposition analysis. The matrix of colored dots indicates specific combinations of driving factors. Yellow, gray, blue and red dots refer to the changes in population, age structure, death rate and $PM_{2.5}$ concentration, respectively. *t0* and *t* refer to the base year (2000 in this study) and target year (from 2001 to 2016). $DAPP_{t0}$ is the annual deaths attributable to $PM_{2.5}$ in the base year, which were calculated based on the factors in the base year. A_t, B_t and C_t are the intermediate variables, which consider the changes in population, age structure and death rate incrementally from the base year to target year. $DAPP_t$, is the annual deaths attributable to $PM_{2.5}$ in the target year, which consider all the changes in four factors. In detailed equations, $PAF_{a,d}$ refers to the disease and age-specific proportion of deaths attributed to $PM_{2.5}$ pollution; POP refers to the total population; $Rate_{a,d}$ is the disease and age-specific death rate; $AgeP_a$ is the proportion of the population with age *a*.

Supplementary Table 1. Comparative summary of study area, exposure-response functions, and input data between previous studies.

Previous study	Region	Involved diseases	Exposure-response function	Input data			
				$PM_{2.5}$	Death rates	Population	Age structure
Xie et al., (2016)[1]	China	Stroke, IHD, LC, COPD	IER from Burnett et al. (2014)[2]	Gridded data from van Donkelaar et al. (2015)[3]	Provincial data from GBD online database[4]	Gridded data from Yang et al. (2009)[5]	Didn't consider specific age-group
Liu et al., (2017)[6]	China	Stroke, IHD, LC	IER from Burnett et al. (2014)[2]	Gridded data from Ma et al. (2016)[7]	National data from China Health Statistical Yearbook[8]	Gridded data from Yang et al. (2009)[5]	Didn't consider specific age-group
Huang et al., (2018)[9]	74 key cities in China	Stroke, IHD, LC, COPD	IER from Burnett et al. (2014)[2]	Monitoring data from China's National Urban Air Quality Real Time Publishing Platform[10]	Regional data estimated based on China Centers for Disease Control and Prevention Dataset and China Death Surveillance Dataset	City level data from China City Statistic Yearbook[11]	City level data from China's population census in 2010[12]
Zou et al., (2019)[13]	China	Stroke, IHD, LC, COPD, LRI	IER from Burnett et al. (2014)[2]	Gridded data estimated based on aerosol optical depth and other auxiliary data	Provincial data estimated based on China Health Statistical Yearbook[8] and Zhou et al. (2016)[14]	Gridded data estimated based on Resource and Environment Science Dataset[15] and China Statistic Yearbook[16]	City level data estimated based on China Statistic Yearbook[16] and China's population census[12]
Lu et al., (2019)	China	Stroke, IHD, LC, COPD	IER from Burnett et al. (2014)[2]	Gridded data estimated based on aerosol optical depth	Provincial data estimated based on GBD online database[4] and Zhou et al. (2016)[14]	Gridded data estimated based on LandScan dataset[17] and China Statistic Yearbook[16]	National data from World Population Prospects Dataset[18]
Guan et al., (2019)	338 cities in China	Stroke, IHD, LC, COPD	IER from Burnett et al. (2014)[2]	Monitoring data from China's National Urban Air Quality Real Time Publishing Platform[10]	Provincial data estimated based on China Death Cause Monitoring Dataset and Zhou et al. (2016)[14]	City level data from multi-level statistic Yearbook[11,16]	Didn't clearly mentioned in the paper
GBD 2017 Risk Factor Collaborators, (2018)	China	Stroke, IHD, LC, COPD, LRI, DM2	IER updated in GBD 2017	Gridded data from Shaddick et al. (2018)[19]	Provincial data from GBD 2017 Causes of Death Collaborators, (2018)[20]	Gridded data from Gridded Population of the World Dataset[21]	National data from GBD online database[4]

Note: The corresponding results can be found in Supplementary Figure 1, and the background color corresponds to the graph legend. IHD, COPD, LC, LRI, and DM2 refer to the ischemic heart disease, chronic obstructive pulmonary disease, lung cancer, lower respiratory infection, and diabetes mellitus type 2, respectively. GBD and IER refers to the Global Burden of Disease and Integrated Exposure-Response function, respectively.

Supplementary Table 2. Comparison between deaths attributable to $PM_{2.5}$ pollution (DAPP) estimated based on national death rates and provincial death rates in 2017.

Province	DAPP estimated based on national death rates (IHD + stroke + COPD + LC)	DAPP estimated based on provincial death rates (IHD + stroke + COPD + LC)	Relative difference in DAPP when calculated using provincial vs national death rates
Beijing	12302	5335	56.6%
Hebei	71721	69462	3.2%
Sichuan	76203	71548	6.1%

Note: Zhou et al. (2019)[22] reported the provincial death rates in 2017 in China. However, these data are only accessible for four diseases (IHD, stroke, COPD and LC) in three provinces (Beijing, Hebei and Sichuan). Hence, we only compared the results that can be estimated by these available data. IHD, COPD, LC refer to ischemic heart disease, chronic obstructive pulmonary disease and lung cancer, respectively. The relative difference was calculated by $(DAPP_{\text{national death rates}} - DAPP_{\text{provincial death rates}}) / DAPP_{\text{national death rates}}$.

Supplementary Note 1. Detailed uncertainty analysis.

Although we considered the 90% confidential intervals of the Integrated Exposure-Response (IER) function as a major source of uncertainty, there are several other uncertainties in our estimation and decomposition of deaths attributable to $PM_{2.5}$ pollution (DAPP). First, the input data might lead to some error and bias. Regarding the estimation of $PM_{2.5}$ concentration, the $PM_{2.5}$ concentration data derived from satellite imagery and modeling tends to be lower than the monitoring data[23]. Although the concentration data in our research was further calibrated by monitoring data, our figures may still be conservative. In addition, at a 10km grid cell spatial resolution, the $PM_{2.5}$ data is relatively coarse which could also lead to underestimation[24]. Besides, we used the annual average $PM_{2.5}$ concentration to express the level of exposure directly, without considering the influence of behavior (inhalation rate and outdoor time) on personal exposure[13,25]. Regarding other input data, especially death rates and age structure, we assumed that the death rates and age structure were homogeneous across the country, because of the accessibility of data. This may also lead to some generalization in the estimation of DAPP at the provincial level. To assess this impact, we compared our estimates and the DAPP derived from provincial death rates using data available only for three provinces (Beijing, Hebei and Sichuan) in 2017, with other inputs fixed. The comparison showed that the use of national death rates in our study led to an overestimate of deaths for Beijing, Hebei and Sichuan. Specifically, for Beijing which has a much better healthcare standard, the DAPP estimated based on provincial death rates was more than 50% lower relative to our estimation based on national death rates. Besides, the differences are relatively minor for Hebei and Sichuan which represent a medium healthcare standard in China. The DAPP estimated based on provincial death rates are 3.2% and 6.1% lower than our main results, respectively[22] (Supplementary Table 2).

Second, the exposure-response relationship between $PM_{2.5}$ concentration and the risk of diseases has some underlying assumptions. For example, the toxicity of $PM_{2.5}$ is assumed to be remain constant with different $PM_{2.5}$ composition. The confounding effects among the different risk factors (e.g., smoking and alcohol use) are not considered, and the relative risk was quantified in terms of annual average $PM_{2.5}$ concentration without considering the short-term variation[2,26]. For $PM_{2.5}$ concentration in China, the proportion of black carbon is higher than the global average[27]. As the toxicity of black carbon is higher than other $PM_{2.5}$ components[28], DAPP tends to be underestimated. The fitting strategy of the exposure-response relationship will also influence our estimation. Although the IER function is widely used, there are also other exposure-response functions available (e.g., global exposure mortality model, GEMM)[29]. We have compared the results between our estimates and the estimates derived from the GEMM, while keeping other inputs constant. The comparison showed that DAPP declined after 2013 based upon both exposure-response functions. The alternative function (GEMM) yielded a larger decline in DAPP, however our key conclusion that China will require stronger policy would still hold (Supplementary Figure 4).

Third, the selection of diseases may introduce uncertainty. We only considered 6 diseases in our estimation, which could lead to an underestimation of DAPP as $PM_{2.5}$ pollution is related to a range of other diseases including mental health[30], Alzheimer's disease[31] and other non-communicable diseases[29]. In addition, because we did not consider the potential double-counting of the deaths arising from household $PM_{2.5}$ pollution, this may introduce some bias in our results[32].

Supplementary Note 2. Detailed description of $PM_{2.5}$ concentration data sources.

In this research, we used the gridded $PM_{2.5}$ concentration from the EFPMV4CH02 dataset (available from 2000–2016)[33], as well as the monitoring data on $PM_{2.5}$ concentration from China's National Urban Air Quality Real Time Publishing Platform (available from 2013–2017)[10]. The gridded $PM_{2.5}$ data was generated by combining the chemical transport model, remotely sensed data and monitoring data in China[34]. The data provider of the gridded $PM_{2.5}$ concentration extends these values back to 2000 using the interannual calibration between the monitoring data and remotely sensed $PM_{2.5}$ data[3]. By combining these data sources, the gridded $PM_{2.5}$ data is effective in both coverage and accuracy (Supplementary Figure 5). We validated the gridded $PM_{2.5}$ concentration dataset by comparing it to monitoring station records from 2013 to 2016 and the gridded $PM_{2.5}$ concentrations showed good agreement with in-situ observations (Supplementary Figure 6). The good agreement between gridded data and monitoring data enabled us to use the 2017 monitoring station $PM_{2.5}$ concentration to extrapolate a gridded $PM_{2.5}$ concentration layer for 2017 (see Supplementary Note 3).

Supplementary Note 3. Estimating gridded $PM_{2.5}$ concentration for 2017.

Gridded $PM_{2.5}$ concentration for 2017 was not available. To fill this data gap, we combined the gridded $PM_{2.5}$ concentration from 2000 to 2016[35] and monitoring data from more than 1000 sites from 2013 to 2017[10], to estimate the gridded $PM_{2.5}$ concentration for 2017 in China (Supplementary Figure 5). We used spatially interpolated monitoring station $PM_{2.5}$ concentration data to extrapolate the 2016 gridded $PM_{2.5}$ concentration layer to create a 2017 gridded $PM_{2.5}$ concentration layer using the following calculation:

$$\mathrm{GC}_t = \mathrm{RMC}_{t,t-1} \times \mathrm{GC}_{t-1}$$

where the GC_t, and GC_{t-1}, refers to the gridded $PM_{2.5}$ concentration in the year t and $t-1$. $RMC_{t,t-1}$ refers to the ratio of monitoring station $PM_{2.5}$ concentrations between year t and t-1, which was calculated based on records from more than 1,000 monitoring stations and spatially interpolated to the pixel scale. Here t = 2017. We validated the abovementioned extrapolation method by extrapolating the population-weighted $PM_{2.5}$ concentration for 2016 using the same method (i.e. letting t = 2016) and validated it against the actual gridded $PM_{2.5}$ concentration data for 2016. The result at prefectural level cities indicated that the estimated population-weighted $PM_{2.5}$ concentration showed good agreement with the gridded dataset (Supplementary Figure 7).

Supplementary Note 4. Calculating deaths attributable to $PM_{2.5}$ pollution at the pixel scale.

Using an established method[32,36-38], we calculated the DAPP in China from 2000 to 2017 at the pixel scale (Supplementary Figure 8). The equation is given as:

$$\mathrm{DAPP}_{i,t} = \sum_{a,d} \left(\mathrm{PAF}_{a,d,i,t} \times \mathrm{POP}_{i,t} \times \mathrm{Rate}_{a,d,t} \times \mathrm{AgeP}_{a,t}\right)$$

where $DAPP_i$ is the deaths attributable to $PM_{2.5}$ pollution for pixel i in year t; $PAF_{a,d,i,t}$ refers to the proportion of deaths attributed to $PM_{2.5}$ pollution caused by disease d in a population with age a at pixel i in year t; and $POP_{i,t}$ refers to the population at pixel i in year t; $Rate_{a,d,t}$ is the death rate of disease d for people with age a, and the $AgeP_{a,t}$ means the percentage of population with age a to the total population in year t. In our estimation, six kinds of diseases related to $PM_{2.5}$ pollution were considered in this study, including lung cancer, chronic obstructive pulmonary disease, lower respiratory infection, ischemic heart disease, stroke, and diabetes mellitus type 2. Fifteen age groups were included in the equation, i.e., 25-30, 30-35…90-95, and beyond 95 years old. For lower respiratory infection, children less than 5 years old was also considered. Because only the national death rates and age structure data were published in China, we assumed that the death rates and age structure were homogeneous across the country, following previous research[39,40].

$PAF_{a,d,i,t}$ was calculated as[38,41,42]:

$$\mathrm{PAF}_{a,d,i,t} = \frac{\mathrm{RR}_{a,d,i,t} - 1}{\mathrm{RR}_{a,d,i,t}}$$

where $RR_{a,d,i,t}$ is the relative risk for the population with age a and have the disease d at pixel i in year t. $RR_{a,d,i,t}$ can be calculated using the integrated exposure-response (IER) function updated in the Global Burden of Disease (GBD) 2017[4,32,36]:

$$\begin{cases} \mathrm{RR}_{a,d,i,t} = 1 + \alpha_{a,d}\left[1 - exp\left(-\gamma_{a,d}\left(\mathrm{C}_{i,t} - \mathrm{C_0}_{a,d}\right)^{\delta_{a,d}}\right)\right], \mathrm{C}_{i,t} > \mathrm{C_0}_{a,d} \\ RR_{a,d,i,t} = 1\ , \qquad \mathrm{C}_{i,t} \le \mathrm{C_0}_{a,d} \end{cases}$$

where $C_{i,t}$ is the $PM_{2.5}$ concentration at pixel *i* in year *t*, and the unit is μg m^{-3}. $\alpha_{a,d}$, $\gamma_{a,d}$, $\delta_{a,d}$, and $C_{0\,a,d}$ are the parameters of the IER model. These parameters were fitted based on multiple epidemiological studies. Specific IER models were used for a given age *a* and a given disease *d*[2]. Since the fitting of the IER model has some uncertainty, following previous studies, the central estimate and 90% uncertainty intervals were obtained as the 50, 5 and 95 percentiles from 1000 draws of each parameter for the IER fitting[38]. For ischemic heart disease and stroke the IERs were age-specific, but for other diseases the IERs were uniform across different age groups.

In GBD 2017, the PAF of ambient $PM_{2.5}$ pollution was calculated using a proportional approach, which used the IER to calculate the relative risk and PAF for both exposure to ambient $PM_{2.5}$ pollution and indoor $PM_{2.5}$ pollution. These were then weighted by the proportion of individuals exposed to each source, in order to avoid the potential double-counting of the health burden that may arise from $PM_{2.5}$ pollution from ambient and household sources[32]. In our study, because of the lack of corresponding indoor $PM_{2.5}$ pollution data, we used the method used in previous GBD reports to calculate the PAF of ambient $PM_{2.5}$ pollution individually[36,37,43,44].

The pixel-level population $POP_{i,t}$ was obtained by allocating the statistical population data of each prefectural-level city. We used the population distribution of the History Database of the Global Environment (HYDE3.2) dataset[45] as the reference data and allocated the statistical population data[11] to each pixel at a spatial resolution of 10km. The process can be expressed as:

$$\mathrm{POP}_{i,t} = \mathrm{HYDEPOP}_{i,t} \times \frac{\mathrm{SPOP}_{p,t}}{\sum_p\left(\mathrm{HYDEPOP}_{i,p,t}\right)}$$

where $POP_{i,t}$ refers to the population at pixel i in year t; $HYDEPOP_{i,t}$ refers to the population at pixel i in the HYDE3.2 dataset in year t; $SPOP_{p,t}$ refers to the statistical population of prefectural-level city p in year t; $HYDEPOP_{i,p,t}$ is the population at pixel i within the prefectural-level city p in the HYDE3.2 dataset in year t. Because some prefectural-level cities did not have statistical population data, we allocated their population in a similar way by using the statistical population data at the provincial level[16].

Supplementary Note 5. Detailed formula of sequential Mann-Kendall test.

The sequential Mann-Kendall test is a nonparametric test, which is widely used in meteorological and hydrological research in detecting abrupt changes in trend[46-48]. The null hypothesis of the sequential Mann-Kendall test is that the elements show no beginning of a developing trend. In the sequential Mann-Kendall test[46], the abrupt point (beginning of a developing trend) in a given series X with n samples can be detected as the intersection point of the test statistics $u_p(t)$ and $u_r(t)$, which are calculated based on the progressive row $\{x_1, x_2, x_3\ldots, x_n\}$ and retrograde row $\{x_n, x_{n-1}, x_{n-2}\ldots, x_1\}$ of the sample, respectively.

The $u_p(t)$ of a given series can be calculated as:

$$u_p(t) = \frac{a_t - E(a_t)}{\sqrt{VAR(a_t)}}$$

where a_t is an auxiliary statistic variable for the point t (varied from 2 to n). a_t is assumed to be asymptotically normal, with a mean value $E(a_t)$ and variance $VAR(a_t)$. These variables can be calculated as:

$$a_t = \sum_{i=1}^{t} R_t$$

$$E(a_t) = \frac{l_t \times (l_t - 1)}{4}$$

$$VAR(a_t) = \frac{l_t \times (l_t - 1) \times (2l_t + 5)}{72}$$

where R_t is the number of precedent elements x_j ($j < t$) that are smaller than x_t, with $t = 2, \ldots, n$, and $j = 1, \ldots, i{-}1$; l_t is the count of elements from x_1 to x_t.

The $u_p(a_t)$ can be calculated using the similar equation, but using a reversed data series $\{x_n, x_{n-1}, x_{n-2}\ldots, x_1\}$ and multiplied by −1.

The point where the test statistics $u_p(t)$ and $u_r(t)$ cross marks the abrupt change in trend. If the

absolute value of $u_p(t)$ and $u_r(t)$ is higher than the tipping point of the given significance level (1.28 for P=0.10 and 1.65 for P=0.05), the null hypothesis will be rejected, which indicates an abrupt change in trend[47]. The intersection point of the test statistics $u_p(t)$ and $u_r(t)$ can be detected as follows:

$$f(x) = \begin{cases} yes, & [u_p(t) - u_r(t)] \times [u_p(t-1) - u_r(t-1)] \leq 0 \\ no, & otherwise \end{cases}$$

where the $u_p(t)$ and $u_r(t)$ represent the test values of the progressive and retrograde row, respectively.

Supplementary References:

1 Xie, R. *et al.* Long-term trend and spatial pattern of PM2.5 induced premature mortality in China. *Environment International* **97**, 180-186 (2016).

2 Burnett, R. *et al.* An Integrated Risk Function for Estimating the Global Burden of Disease Attributable to Ambient Fine Particulate Matter Exposure. *Environmental Health Perspectives* **122**, 397-403 (2014).

3 van Donkelaar, A., Martin, R., Brauer, M. & Boys, B. Use of Satellite Observations for Long-term Exposure Assessment of Global Concentrations of Fine Particulate Matter. *Environmental Health Perspectives* **123**, 135-143 (2015).

4 Institute for Health Metrics and Evaluation. GBD Compare. https://vizhub.healthdata.org/gbd-compare/ (2018).

5 Yang, X., Huang, Y., Dong, P., Jiang, D. & Liu, H. An Updating System for the Gridded Population Database of China Based on Remote Sensing, GIS and Spatial Database Technologies. *Sensors* **9**, 1128-1140 (2009).

6 Liu, M. *et al.* Spatial and temporal trends in the mortality burden of air pollution in China: 2004-2012. *Environment international* **98**, 75-81 (2017).

7 Ma, Z. *et al.* Satellite-Based Spatiotemporal Trends in PM 2.5 Concentrations: China, 2004–2013. *Environmental Health Perspectives* **124**, 184-192 (2016).

8 National Health and Family Planning Commission of China. China Health Statistical Yearbook. http://navi.cnki.net/knavi/YearbookDetail?pcode=CYFD&pykm=YSIFE (2013).

9 Huang, J., Pan, X., Guo, X. & Li, G. Health impact of China's Air Pollution Prevention and Control Action Plan: an analysis of national air quality monitoring and mortality data. *The Lancet Planetary Health* **2**, e313-e323 (2018).

10 Ministry of Ecology and Environment of China. China's National Urban Air Quality Real Time Publishing Platform. http://www.mee.gov.cn/hjzl/dqhj/qgkqzlssfb/ (2013).

11 National Bureau of Statistics of China. China City Statistics Yearbooks. http://navi.cnki.net/knavi/YearbookDetail?pcode=CYFD&pykm=YINFN (2018).

12 National Bureau of Statistics of China. The sixth population census of China. http://www.stats.gov.cn/tjsj/pcsj/rkpc/6rp/indexch.htm (2011).

13 Zou, B. *et al.* Air pollution intervention and life-saving effect in China. *Environment International* **125**, 529-541 (2019).

14 Zhou, M. *et al.* Cause-specific mortality for 240 causes in China during 1990–2013: a

systematic subnational analysis for the Global Burden of Disease Study 2013. *The Lancet* **387**, 251-272 (2016).

15 Chinese Academy of Science. Resource and Environment Science Data Center. http://www.resdc.cn/ (2019).

16 National Bureau of Statistics of China. China Statistics Yearbooks. http://navi.cnki.net/knavi/YearbookDetail?pcode=CYFD&pykm=YZGCA (2019).

17 Rose, A. N., McKee, J. J., Urban, M. L. & Bright, E. A. LandScan 2017. https://landscan.ornl.gov/ (2018).

18 United Nations. World Population Prospects 2019. https://population.un.org/wpp/Download/Standard/Population/ (2019).

19 Shaddick, G. *et al.* Data integration model for air quality: a hierarchical approach to the global estimation of exposures to ambient air pollution. *Journal of the Royal Statistical Society Series C-Applied Statistics* **67**, 231-253 (2018).

20 GBD 2017 Causes of Death Collaborators. Global, regional, and national age-sex-specific mortality for 282 causes of death in 195 countries and territories, 1980-2017: a systematic analysis for the Global Burden of Disease Study 2017. *The Lancet* **392**, 1736-1788 (2018).

21 Center for International Earth Science Information Network. Gridded Population of the World, Version 4 (GPWv4): Population Count, Revision 10. https://doi.org/10.7927/H4PG1PPM (2017).

22 Zhou, M. *et al.* Mortality, morbidity, and risk factors in China and its provinces, 1990–2017: a systematic analysis for the Global Burden of Disease Study 2017. *The Lancet* **394**, 1154-1158 (2019).

23 Jerrett, M. *et al.* Comparing the Health Effects of Ambient Particulate Matter Estimated Using Ground-Based versus Remote Sensing Exposure Estimates. *Environmental Health Perspectives* **125**, 552-559 (2017).

24 Li, Y., Henze, D. K., Jack, D. & Kinney, P. L. The influence of air quality model resolution on health impact assessment for fine particulate matter and its components. *Air Quality, Atmosphere & Health* **9**, 51-68 (2016).

25 Zou, B. *et al.* Efforts in reducing air pollution exposure risk in China: State versus individuals. *Environment International* **137**, https://doi.org/10.1016/j.envint.2020.105504 (2020).

26 Liu, C. *et al.* Ambient Particulate Air Pollution and Daily Mortality in 652 Cities. *New England Journal of Medicine* **381**, 705-715 (2019).

27 Philip, S. *et al.* Global Chemical Composition of Ambient Fine Particulate Matter for

Exposure Assessment. *Environmental Science & Technology* **48**, 13060-13068 (2014).

28 Lelieveld, J., Evans, J. S., Fnais, M., Giannadaki, D. & Pozzer, A. The contribution of outdoor air pollution sources to premature mortality on a global scale. *Nature* **525**, 367-371 (2015).

29 Burnett, R. *et al.* Global estimates of mortality associated with long-term exposure to outdoor fine particulate matter. *Proceedings of the National Academy of Sciences* **115**, 9592-9597 (2018).

30 Zhang, X., Chen, X. & Zhang, X. The impact of exposure to air pollution on cognitive performance. *Proceedings of the National Academy of Sciences* **115**, 9193-9197 (2018).

31 Underwood, E. The polluted brain: Evidence builds that dirty air causes A lzheimer's, dementia. http://www.sciencemag.org/news/2017/01/brain-pollution-evidence-builds-dirty-air-causes-alzheimer-s-dementia (2017).

32 GBD 2017 Risk Factor Collaborators. Global, regional, and national comparative risk assessment of 84 behavioural, environmental and occupational, and metabolic risks or clusters of risks for 195 countries and territories, 1990–2017: a systematic analysis for the Global Burden of Disease Study 2017. *The Lancet* **392**, 1923-1994 (2018).

33 Atmospheric Composition Analysis Group. Surface PM2.5,. http://fizz.phys.dal.ca/~atmos/martin/?page_id=140 (2019).

34 van Donkelaar, A., Martin, R. V., Li, C. & Burnett, R. T. Regional Estimates of Chemical Composition of Fine Particulate Matter Using a Combined Geoscience-Statistical Method with Information from Satellites, Models, and Monitors. *Environmental Science & Technology* **53**, 2595-2611 (2019).

35 van Donkelaar, A. *et al.* Global Estimates of Fine Particulate Matter using a Combined Geophysical-Statistical Method with Information from Satellites, Models, and Monitors. *Environ Sci Technol* **50**, 3762-3772 (2016).

36 Cohen, A. *et al.* Estimates and 25-year trends of the global burden of disease attributable to ambient air pollution: an analysis of data from the Global Burden of Diseases Study 2015. *The Lancet* **389**, 1907-1918 (2017).

37 GBD 2016 Risk Factors Collaborators. Global, regional, and national age-sex specific mortality for 264 causes of death, 1990-2016: a systematic analysis for the Global Burden of Disease Study 2016. *The Lancet* **390**, 1151-1210 (2017).

38 World Health Organization. Ambient air pollution: A global assessment of exposure and burden of disease. http://www.who.int/phe/publications/air-pollution-global-assessment/en/ (2016).

39 Silva, R., Adelman, Z., Fry, M. & West, J. The Impact of Individual Anthropogenic Emissions Sectors on the Global Burden of Human Mortality due to Ambient Air Pollution.

Environmental Health Perspectives **124**, 1776-1784 (2016).

40 Liu, J., Han, Y., Tang, X., Zhu, J. & Zhu, T. Estimating adult mortality attributable to PM2.5 exposure in China with assimilated PM2.5 concentrations based on a ground monitoring network. *Science of The Total Environment* **568**, 1253-1262 (2016).

41 Rockhill, B., Newman, B. & Weinberg, C. Use and misuse of population attributable fractions. *American Journal of Public Health* **88**, 15-19 (1998).

42 World Health Organization. Metrics: Population Attributable Fraction (PAF). http://www.who.int/healthinfo/global_burden_disease/metrics_paf/en/ (2017).

43 GBD 2013 Risk Factors Collaborators. Global, regional, and national comparative risk assessment of 79 behavioural, environmental and occupational, and metabolic risks or clusters of risks in 188 countries, 1990–2013: a systematic analysis for the Global Burden of Disease Study 2013. *The Lancet* **386**, 2287-2323 (2015).

44 GBD MAPS Working Group. Burden of Disease Attributable to Coal-Burning and Other Air Pollution Sources in China. https://www.healtheffects.org/publication/burden-disease-attributable-coal-burning-and-other-air-pollution-sources-china (2016).

45 Goldewijk, K., Beusen, A., Doelman, J. & Stehfest, E. New anthropogenic land use estimates for the Holocene; HYDE 3.2. *Earth System Science Data Discussions*, 1-40 (2016).

46 World Meteorological Organization. On the statistical analysis of series of observations. https://library.wmo.int/index.php?lvl=notice_display&id=7427#.XWUz2egzaM8 (1991).

47 Gerstengarbe, F.-W. & Werner, P., C. . Estimation of the beginning and end of recurrent events within a climate regime. *Climate Research* **11**, 97-107 (1999).

48 Yang, Y. & Tian, F. Abrupt change of runoff and its major driving factors in Haihe River Catchment, China. *Journal of Hydrology* **374**, 373-383 (2009).
