# Supplementary material for: Stronger policy required to substantially reduce deaths from PM2.5 pollution in China
Source: Nat Commun. 2020 Mar 19;11:1462. doi: 10.1038/s41467-020-15319-4 (PMC7081205; doi:10.1038/s41467-020-15319-4)
Supplement: Supplementary file 2 — Reporting Summary [file 41467_2020_15319_MOESM2_ESM.pdf]

nature research

Corresponding author(s): Chunyang He
Last updated by author(s): Mar 3, 2020

Reporting Summary

Nature Research wishes to improve the reproducibility of the work that we publish. This form provides structure for consistency and transparency in reporting. For further information on Nature Research policies, see Authors & Referees and the Editorial Policy Checklist.

Statistics

For all statistical analyses, confirm that the following items are present in the figure legend, table legend, main text, or Methods section.

n/a	Confirmed	
☒	☐	The exact sample size (*n*) for each experimental group/condition, given as a discrete number and unit of measurement
☒	☐	A statement on whether measurements were taken from distinct samples or whether the same sample was measured repeatedly
☐	☒	The statistical test(s) used AND whether they are one- or two-sided *Only common tests should be described solely by name; describe more complex techniques in the Methods section.*
☒	☐	A description of all covariates tested
☒	☐	A description of any assumptions or corrections, such as tests of normality and adjustment for multiple comparisons
☐	☒	A full description of the statistical parameters including central tendency (e.g. means) or other basic estimates (e.g. regression coefficient) AND variation (e.g. standard deviation) or associated estimates of uncertainty (e.g. confidence intervals)
☒	☐	For null hypothesis testing, the test statistic (e.g. *F*, *t*, *r*) with confidence intervals, effect sizes, degrees of freedom and *P* value noted *Give P values as exact values whenever suitable.*
☒	☐	For Bayesian analysis, information on the choice of priors and Markov chain Monte Carlo settings
☒	☐	For hierarchical and complex designs, identification of the appropriate level for tests and full reporting of outcomes
☒	☐	Estimates of effect sizes (e.g. Cohen's *d*, Pearson's *r*), indicating how they were calculated

Our web collection on statistics for biologists contains articles on many of the points above.

Software and code

Policy information about availability of computer code

Data collection	No code or software was used in data collection.
Data analysis	We used the integrated exposure-response function updated in the Global Burden of Disease 2017 to estimate the relative risk caused by PM2.5 exposure. The detailed function is accessible to all users at http://ghdx.healthdata.org/record/ihme-data/gbd-2017-burden-risk-1990-2017. Other data processing in this study are conducted at ArcGIS and Microsoft Excel

For manuscripts utilizing custom algorithms or software that are central to the research but not yet described in published literature, software must be made available to editors/reviewers. We strongly encourage code deposition in a community repository (e.g. GitHub). See the Nature Research guidelines for submitting code & software for further information.

Data

Policy information about availability of data

All manuscripts must include a data availability statement. This statement should provide the following information, where applicable:

- Accession codes, unique identifiers, or web links for publicly available datasets
- A list of figures that have associated raw data
- A description of any restrictions on data availability

All the data created in this study are openly available at https://github.com/yuehuanbi/air-pollution. Other data are available from the corresponding author upon reasonable request.

Field-specific reporting

Please select the one below that is the best fit for your research. If you are not sure, read the appropriate sections before making your selection.

☐ Life sciences ☐ Behavioural & social sciences ☒ Ecological, evolutionary & environmental sciences

For a reference copy of the document with all sections, see nature.com/documents/nr-reporting-summary-flat.pdf

Ecological, evolutionary & environmental sciences study design

All studies must disclose on these points even when the disclosure is negative.

Study description	We estimated the annual deaths attributable to ambient PM2.5 pollution in China from 2000 to 2017 at a pixel scale (10km) by combining an epidemiological model and long term PM2.5 data.
Research sample	We used age- and disease specific death rate data from Global Burden of Disease 2017. Six kinds of diseases related to PM2.5 pollution were considered in this study, including lung cancer, chronic obstructive pulmonary disease, lower respiratory infection, ischemic heart disease, stroke, and diabetes mellitus type 2. Fifteen age groups were included in the equation, i.e., 25–30, 30–35… 90–95, and beyond 95 years old. For lower respiratory infection, children less than 5 years old were also considered.
Sampling strategy	The age group and disease were chosen following the widely used Global Burden of Disease study.
Data collection	The PM2.5 data, demographic data and death rate data can be download directly. Detailed links were supported in the Method section.
Timing and spatial scale	The PM2.5 data are available annually from 2000 to 2016, with a resolution of 0.01 degrees. The PM2.5 concentration in 2017 was extrapolated based on the monitoring data. Population distribution data contained the population from 2000 to 2017 with a spatial resolution of 0.083 degrees. Data on the age structure of China's population from 2000 and 2017, as well as the age- and disease-specific death rates were obtained from the Global Burden of Disease 2017 dataset, at a national scale.
Data exclusions	No data were excluded in this study.
Reproducibility	All attempts to repeat the experiment were successful.
Randomization	This is not applicable in this study
Blinding	Blinding is not applicable in this study

Did the study involve field work? ☐ Yes ☒ No

Reporting for specific materials, systems and methods

We require information from authors about some types of materials, experimental systems and methods used in many studies. Here, indicate whether each material, system or method listed is relevant to your study. If you are not sure if a list item applies to your research, read the appropriate section before selecting a response.

Materials & experimental systems

n/a	Involved in the study
☒	☐ Antibodies
☒	☐ Eukaryotic cell lines
☒	☐ Palaeontology
☒	☐ Animals and other organisms
☒	☐ Human research participants
☒	☐ Clinical data

Methods

n/a	Involved in the study
☒	☐ ChIP-seq
☒	☐ Flow cytometry
☒	☐ MRI-based neuroimaging
